# Colchicine Protects Against Sickle Cell Related Cardiomyopathy: Evidence of the Novel Role of Inflammaging

**DOI:** 10.3390/antiox15080912

**Published:** 2026-07-23

**Authors:** Iana Iatcenko, Enrica Federti, Alessandra Ghigo, Jacopo Ceolan, Rebecca Priolo, Antonio Recchiuti, Immacolata Andolfo, Achille Iolascon, Alessandro Matte, Richard Pozzetto Huot, Veronica Riccardi, Simone Villaboni, Filippo Mazzi, Emanuela Tolosano, Elisa Gremese, Manuela Stella, Gian Luca Forni, Lucia De Franceschi

**Affiliations:** 1 Department of Engineering for Innovative Medicine, University of Verona, 37134 Verona, Italy; 2 Department Molecular Biotechnology and Health Sciences, Molecular Biotechnology Center, University of Torino, 10126 Torino, Italy; alessandra.ghigo@unito.it (A.G.);; 3 Department of Medical, Oral, and Biotechnology Science, “G. d’Annunzio” University of Chieti-Pescara, 66100 Chieti, Italy; 4 Dipartimento Scienze Chimiche, Università Degli Studi di Napoli “Federico II”, 80126 Naples, Italyachille.iolascon@unina.it (A.I.); 5 Centro di Biotecnologie Avanzate “Franco Salvatore”, 80131 Naples, Italy; 6 Rheumatology and Clinical Immunology, IRCCS Humanitas Research Hospital, 20089 Milan, Italy; 7 Department of Biomedical Sciences, Humanitas University, Pieve Emanuele, 20072 Milan, Italy; 8 Department of Internal Medicine, Pharmacology & Toxicology, University of Genova, 16132 Genova, Italy; 9 Servizio Sperimentazioni Cliniche Pediatriche, Istituto Gaslini, 16147 Genova, Italy; 10 UOC Ematologia, IRCCS Istituto G. Gaslini, 16147 Genoa, Italy; 11 FORANEMIA ETS Foundation, 16147 Genoa, Italy; 12 Azienda Ospedaliera Integrata (AOUI) of Verona, 37126 Verona, Italy

**Keywords:** sickle cell disease, cardiomyopathy, inflammasome, Th17, IL17

## Abstract

Sickle cell disease (SCD) is a globally distributed hereditary red cell disorder with still high mortality. Growing evidence indicates that sickle cell related cardiovascular disease contributes to the early death of adults with SCD. Here, we show that humanized SCD mice developed an age-dependent cardiomyopathy characterized by (i) increased circulating Th17 lymphocytes, Th17 heart infiltration associated with increased plasma IL-17; (ii) collagen deposition and activation of both platelet derived growth factor-B (PDGF-B) and transforming growth factor-β1 (TGF-β1) canonical pathways; (iii) overactivation of heart NF-κB associated with up-regulation of NLRP3 and of inflammatory vasculopathy markers. We then used colchicine (CLC) that acts as anti-inflammatory drug with immunomodulatory effects. In humanized SCD mice, we demonstrated the protective role of low-dose CLC treatment against chronic inflammation and preserving myocardial performance. Of note, we found CLC also attenuating sickle cell related lung damage, suggesting a multiorgan effect of CLC in SCD mice. Our data generate the rationale to further explore CLC as new therapeutic tool to treat early stages of sickle cell cardiomyopathy.

## 1. Introduction

Sickle cell disease (SCD) is a worldwide distributed hereditary red cell disorder with still high mortality and limited therapeutic options. Inflammatory vasculopathy and sickle cell related cardiovascular disease has been shown to negatively impact patients’ quality of life and to contribute to early death of adults with SCD [1,2]. Studies in both human and mouse models for SCD suggest the crucial role of chronic mild inflammation or unresolved inflammation combined with local acute ischemic/reperfusion stress in pathologic heart remodeling and cardiac fibrosis [2,3,4,5]. Recently, in humanized mouse model of SCD, we showed the key role of cardiac neutrophil-driven response to ischemic/reperfusion stress in triggering the local overactivation of NF-kB and TGF-β canonical pathway in heart pathologic remodeling [4]. Although the mechanisms involved in the pathogenesis of sickle cell related cardiomyopathy have been explored, much still remains to be investigated on the natural history of sickle cell related cardiomyopathy. Using the “Berk” sickle cell mice, Bakeer et al. showed the important role of chronic mild inflammation in sustaining the activation of pathways involved in matrix deposition, neo-angiogenesis and lipid metabolism, highlighting the limited contribution of chronic anemia in the pathogenesis of sickle cell related cardiomyopathy [5]. Indeed, increased pro-inflammatory and pro-fibrotic IL18 characterized both human and mice with SCD further linking chronic mild inflammation as sterile inflammation and maladaptive heart remodeling [6,7]. The biologic importance of chronic mild inflammation in the pathogenesis of sickle cell related cardiomyopathy is also supported by the observation that early treatment with either hydroxyurea or red cell transfusion delay the development of heart fibrosis in patients with SCD [8]. Studies in different models of cardiac fibrosis highlight the novel role of T-lymphocyte heart recruitment in pathological heart remodeling [9]. Among T-lymphocytes, Th17 subgroup has been recently shown to participate to cardiac fibrosis by sustaining local inflammation and activation of extracellular pathologic heart remodeling [10,11,12]. Indeed, the Th17 cells secrete pro-inflammatory cytokines as IL17, which is also expressed by cardiomyocytes. IL17 promotes local communication between heart neutrophil and lymphocyte infiltrates, contributing to the activation of NF-kB and TGF- b pathways ending with the progression of heart fibrosis [10,11]. Notably, increased IL17 has been associated with cardiac dysfunction and maladaptive heart remodeling, suggesting IL17 as marker for cardiovascular disease progression [11]. In patients with SCD, increase Th17 and plasma IL17 have been reported [13,14,15]. This is associated with markers of inflammatory vasculopathy, pain sensitization and higher risk of early death [13,14,15]. Of note, both Th17 and plasma IL17 are not modulated by acute VOCs [15]. Colchicine (CLC) is a long-used therapeutic agent with anti-inflammatory profile, mainly used to treat gout or Mediterranean fever [16]. CLC has been recently tested in different human and mouse models of cardiomyopathy (e.g.,: acute/chronic coronary disease, atrial fibrillation and pericardial disease), reducing the risk of ischemic events when used in secondary prevention [16]. Indeed, CLC has been reported to protect against ischemic/reperfusion damage, to modulate innate immunity adaptive mechanisms and heart inflammatory cell infiltrates (e.g.,: neutrophil, lymphocytes, Th17). CLC mainly targets NF-kB activation and inflammasome, modulating also the Nrf2 related pathways [17,18,19].

Here, we studied the natural history of sickle cell cardiomyopathy in humanized SS mice. In aging SCD mice, we found that mild persistent inflammation promotes the activation of pro-fibrotic pathways associated with increased plasma IL17 and heart Th17 infiltration in the absence of major changes in neutrophil heart infiltration. CLC protects against sickle cell related cardiomyopathy more efficiently than hydroxyurea (HU), suggesting CLC as interesting novel therapeutic option to delay/interfere with the progression of sickle cell related cardiomyopathy.

## 2. Materials and Methods

### 2.1. Design of the Study

Experiments were carried out using 3 (young adult) and 8 (old-adult) months-old sex and aged matched healthy [Hbatm1(HBA)Tow Hbbtm3(HBG1, HBB)Tow] (AA) and sickle cell [Hbatm1(HBA)Tow Hbbtm2(HBG1,HBB*)Tow] (SS) mice [4]. Here, we focus on 3 and 8 months old healthy and sickle cell mice based on Bakeer et al. and on our experiments showing age-related activation of heart NF-kB pathways in 8-months old healthy animals (see Section 3.2). The Institutional Animal-Experimental-Committee of University of Verona and the Italian Ministry of Health approved the experimental protocols (56DC9.64). The sample size was estimated based on previous studies using humanized SS mice [4]. A double-blind-strategy was implemented to minimize the potential bias in data collection. Animals with either skin lesion or Hb levels < 5 g/dL were excluded. Mice were anesthetized with isoflurane and randomly assigned to experimental groups. Whole blood was collected via retro-orbital venipuncture by heparinized microcapillaries [20]. In anesthetized perfused animals, hearts were rapidly removed and divided into two and either immediately frozen in liquid nitrogen or fixed in 4% paraformaldehyde (O/N at 4 °C). CLC (0.1 mg/kg daily) or vehicle (water) were administrated for 4 months by gavage, starting at 3-4 months of age [21]. The dosage of CLC was based on the review of the literature and the reported beneficial effect of low dose CLC in cardiovascular disease [22,23,24]. A subgroup of animals treated with hydroxyurea (HU) at the dosage of 50 mg/Kg/d for 3 months was also evaluated [25]. Hemoglobin (Hb), liver enzymes (aspartate aminotransferase, AST; alanine aminotransferase, ALT) were determined as previously described [26,27]. Plasma cytokines were carried out using ELLA (Bio-Techne, Minneapolis, MN, USA); plasma pro-BNP was measured by NT-pro-BNP ELISA kit (Nordic BioSite AB, Täby, Sweden). Details on echocardiography, heart histological analysis, flow cytometric analysis of Th17 cells, Th17 heart, spleen infiltrate, heart immunoblot analysis, immunoprecipitation assay, heart molecular analysis are reported in Supplementary Data [3,4,28,29,30,31].

### 2.2. Statistics

Data were presented mean ± standard error of the mean (SEM). Comparison among groups were performed using parametric or non-parametric tests according to normality of distributions assessed using the Shapiro–Wilk test. In the case of two groups, a two-tailed unpaired *t*-test with Welch’s correction or Mann–Whitney U test was used, whereas comparisons involving more than two groups were performed using one-way ANOVA/Weilch’s ANOVA test, followed by multiple comparisons test (Tukey’s or Dunnet’s), or Kruskal–Wallis test followed by Dunn’s multiple comparisons test. Differences with *p* < 0.05 were considered statistically significant. Statistical analyses were performed using GraphPad Prism 10.2. All data are stored in the Nas-Synology-DS216se-Hard-Disk, located at the University of Verona and will be available on request.

## 3. Results

### 3.1. Sickle Cell Mice Display an Age-Dependent Effect on Cardiac Fibrosis Associated with Overactivation of Pro-Fibrotic Pathways

**Figure 1:**
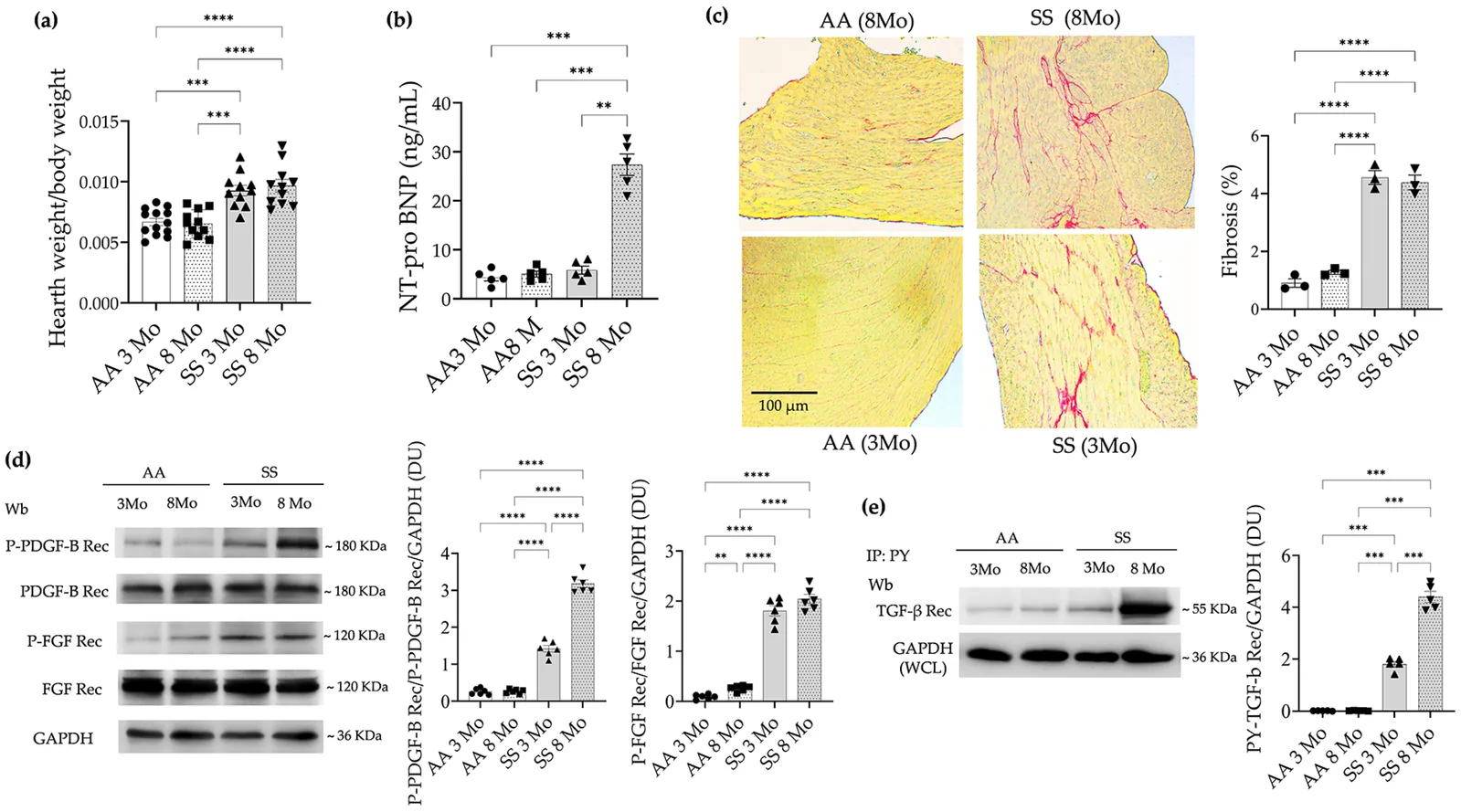
**Sickle cell mice develop an age dependent cardiomyopathy with over-activation of pro-fibrotic pathways and heart fibrosis.** (**a**) Heart weight to body weight ratio in healthy AA mice and sickle cell (SS) mice at 3 and 8 months of age. (**b**) Plasmatic NT-pro-BNP level in healthy (AA) and sickle (SS) mice at 3 and 8 months of age. (**c**) **Left panel**: Picrosirius Red staining in cardiac slices from AA and SS mice at 3 and 8 months of age. **Right panel**: Quantification of heart fibrosis. (**d**) Immunoblot analysis, using specific antibodies against phosphorylated p-PDGFR-B, PDGFR-B, (p)-FGFR, and FGFR, in heart from AA and SS mice as in a. 75 µg/µL of protein loaded on an 8% T, 2.5% C polyacrylamide gel. GAPDH serves as protein loading control. One representative gel from 6 with similar results is shown. Densitometric analysis of immunoblots is shown on the right. (**e**) Immunoprecipitation from heart from AA and SS mice as in (**d**), using specific anti-phospho-Tyrosine antibodies (IP: PY), revealed with specific anti-TGF β receptor (Rec) antibody (75 µg/µL of protein loaded on an 8% T, 2.5%C polyacrylamide gel). GAPDH in whole cell lysate (WCL) is used as loading controls. Data in the column charts are presented as individual data points with mean ± SEM. Significance codes: * *p* < 0.05; ** *p* < 0.01; *** *p* < 0.001; **** *p* < 0.0001; not significant comparisons were not reported. Adjusted *p*-values were used when multiple comparisons were involved.

Bakeer et al. showed a limited effect of anemia on the pathogenesis of sickle cell related cardiomyopathy [5]. In 3- and 8-months of age, SS mice, we found similar degree of anemia, but significantly lower Hb level when compared to healthy animals (Supplementary Figure S1a). 3- and 8-months old SS mice displayed hypertrophic cardiomyopathy, which phenotype was confirmed at both the organ and cellular levels, as evidenced by (i) increased heart weight–to–body weight (HW/BW) ratios; (ii) enlarged cardiomyocyte cross-sectional area; (iii) an age dependent increased in pro-BNP; used as a marker of cardiac dysfunction; (iv) an age dependent increased collagen deposition in heart from 8-months old SS mice when compared to 3-months old SS mice or healthy animals (Figure 1a–c, Supplementary Figure S1b). This was associated with the increased degradation of SERCA2A, sarcoplasmic reticulum calcium ATPase cardiac isoform, a calcium transporter important in myocardial performance in the absence of heart iron accumulation (Supplementary Figure S1c,d). In addition, we found an age dependent activation of PDGF-B receptor and TGF-β receptor, while the FGF-receptor was similarly activated in 3- and 8-months old SS mice when compared to healthy animals (Figure 1d,e).

Taken together these data indicate an age dependent progression of sickle cell-related cardiomyopathy due to overactivation of pro-fibrotic pathways and progressive worsening of cardiac fibrosis.

### 3.2. In Heart from Aging Sickle Cell Mice, the Persistent Pro-Inflammatory Response Is Associated with Heart Recruitment of Lymphocytes Th17 and Increased Plasma IL17

**Figure 2:**
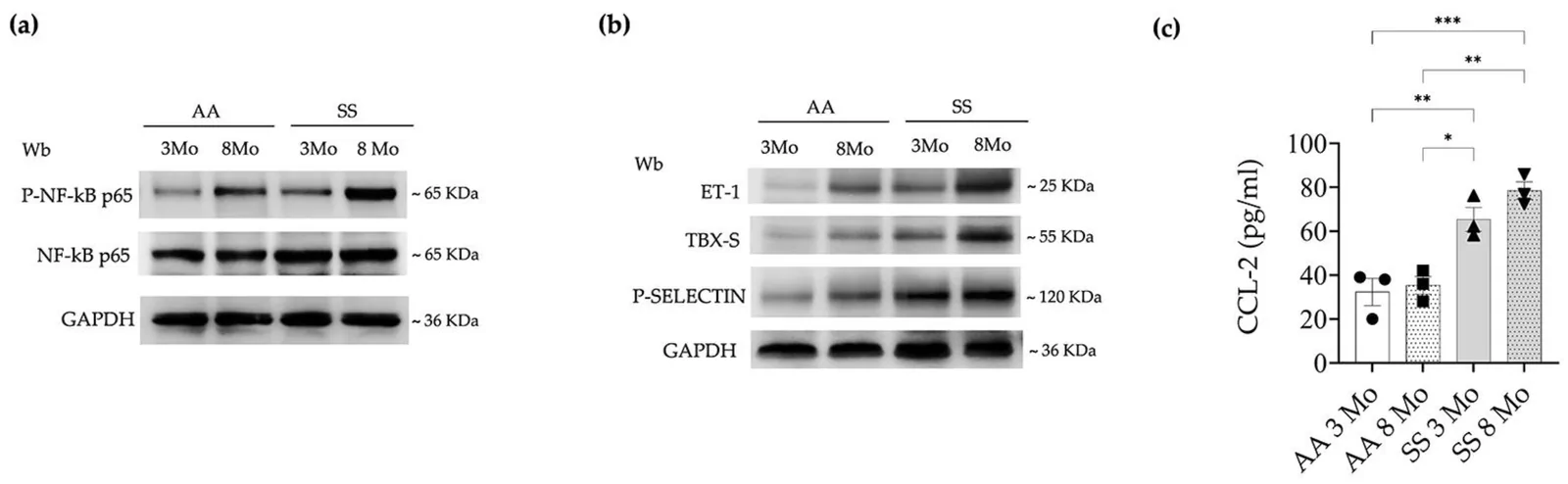
**Sickle cell mice display age dependent heart activation of NF-kB dependent pro-inflammatory pathways.** (**a**) Immunoblot analysis, using specific antibodies against phosphorylated (p)NF-κB p65 and NF-κB p65, of heart from healthy (AA) and sickle cell (SS) mice at 3 and 8 months of age. 75 µg/µL of protein loaded on an 8% T, 2.5%C polyacrylamide gel. GAPDH serves as protein loading control. One representative gel from 6 with similar results is shown. Densitometric analysis of immunoblots is shown in Supplementary Figure S2a. (**b**) Immunoblot analysis, using specific antibodies against endothelin-1 (ET-1), thromboxane synthase (TBXS) and P-Selectin in heart from AA and SS mice as in a. 75 µg/µL of protein loaded on an 11% T, 2.5%C polyacrylamide gel. GAPDH serves as protein loading control. One representative gel from 6 with similar results is shown. Densitometric analysis of immunoblots is shown in Supplementary Figure S2b. (**c**) Plasma chemokine (C-C Motif) Ligand 2 (CCL2) in AA and SS mice as in a. Data in the column charts are presented as individual data points with mean ± SEM. Significance codes: * *p* < 0.05; ** *p* < 0.01; *** *p* < 0.001; **** *p* < 0.0001; not significant comparisons were not reported. Adjusted *p*-values were used when multiple comparisons were involved.

Since we previously showed that NF-kB plays a crucial role in the pathogenesis of cardiac fibrosis in sickle cell mice exposed to hypoxia/reoxygenation stress, we evaluated NF-kB related pathways in hearts from aging animals of both mouse strains [4]. As shown in Figure 2, we found an age dependent activation of NF-kB in hearts from sickle cell mice (Figure 2a, Supplementary Figure S2a).

**Figure 3:**
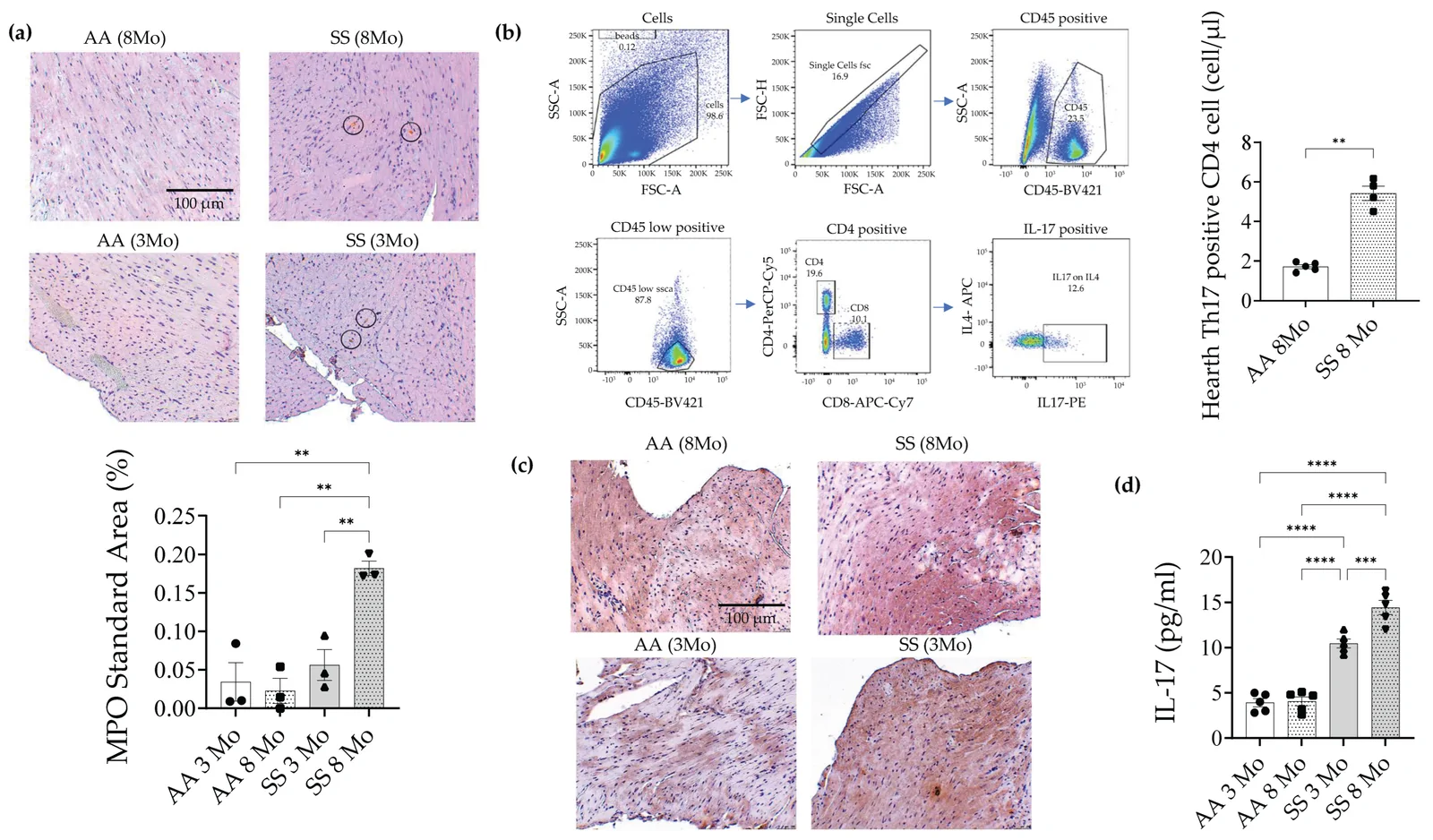
**Sickle cell mice showed neutrophil and lymphocyte Th17 heart infiltration.** (**a**) Representative myeloperoxidase (MPO) immunohistochemical staining of cardiac sections from AA and SS mice at 3 and 8 months of age. MPO quantification is shown in the lower panel. Circles highlight MPO-positive cells. (**b**) **Left panel**: Gating strategy for Th17 heart infiltrates. **Right panel**: Heart IL-17 infiltration identified by flow cytometric analysis in AA and SS mice at 8 months of age. (**c**) Representative IL-17 immunohistochemical staining of cardiac sections from AA and SS mice at 3 and 8 months of age. IL-17 quantification is shown in Supplementary Figure S3a. (**d**) Plasma IL-17 in AA and SS mice as in a. Data in the column charts are presented as individual data points with mean ± SEM. Significance codes: * *p* < 0.05; ** *p* < 0.01; *** *p* < 0.001; **** *p* < 0.0001; not significant comparisons were not reported. Adjusted *p*-values were used when multiple comparisons were involved.

This was associated with age-dependent up-regulation of markers of inflammatory vasculopathy such as endothelin-1 (ET-1) and thromboxane synthase 1 (TBX1) in heart from SCD mice when compared to healthy animals (Figure 2b, Supplementary Figure S2b). Heart P-selectin expression was stably higher in hearts from both 3- and 8- months old SS mice when compared to healthy mice (Figure 2b). Of note, we observed age-dependent activation of NF-kB pathways also in healthy mice but to a lower degree when compared to hearts from SCD animals (Figure 2a,b; Supplementary Figure S2a,b). Taking together these data suggest a local chronic mild inflammation or unresolved inflammation persisting throughout ageing. This was also supported by the persistent increase throughout aging of soluble CCL2 (MCP-1), a pro-inflammatory cytokine identified as risk factor for cardiovascular mortality (Figure 2c) [32,33]. Previous studies highlight the role of CCL2 in heart inflammatory neutrophil/lymphocyte recruitment [34]. In SCD, we previously showed a delay heart neutrophile clearance in SS mice exposed to H/R stress. Thus, we asked whether heart immune infiltration might participate to age-dependent sickle cell related cardiomyopathy. As shown in Figure 3a, we observed increased neutrophil heart infiltration in 8-months old SS mice when compared to either 3-months old SS animals or healthy mice.

We then asked whether other immune cell types might locally crosstalk with heart neutrophils. Indeed, we observed increased Th17 heart infiltration associated with increase heart IL17 expression and soluble IL17 levels when compared to healthy animals (Figure 3b, Supplementary Figure S3). This is interesting since CCL2 might be induced by IL17, a potent pro-inflammatory and pro-fibrotic cytokine involved in heart maladaptive remodeling [34]. In SCD mice, we observed increased circulating and spleen Th17 lymphocytes in SS mice when compared to healthy animals (Supplementary Figure S4).

Collectively, our data indicate that during aging chronic mild inflammation contributes to the development of cardiac fibrosis by the continuum infiltration of neutrophil and Th17 lymphocytes promoted by local IL17 [10,35]. This synergizes with ET-1, a known pro-inflammatory and pro-fibrotic cytokine, which we previously showed to play a key role in the pathogenesis of sickle cell related heart fibrosis [4].

### 3.3. In Sickle Cell Mice, CLC Preserves Myocardial Performance and Protects Against the Activation of Profibrotic Pathways

**Figure 4:**
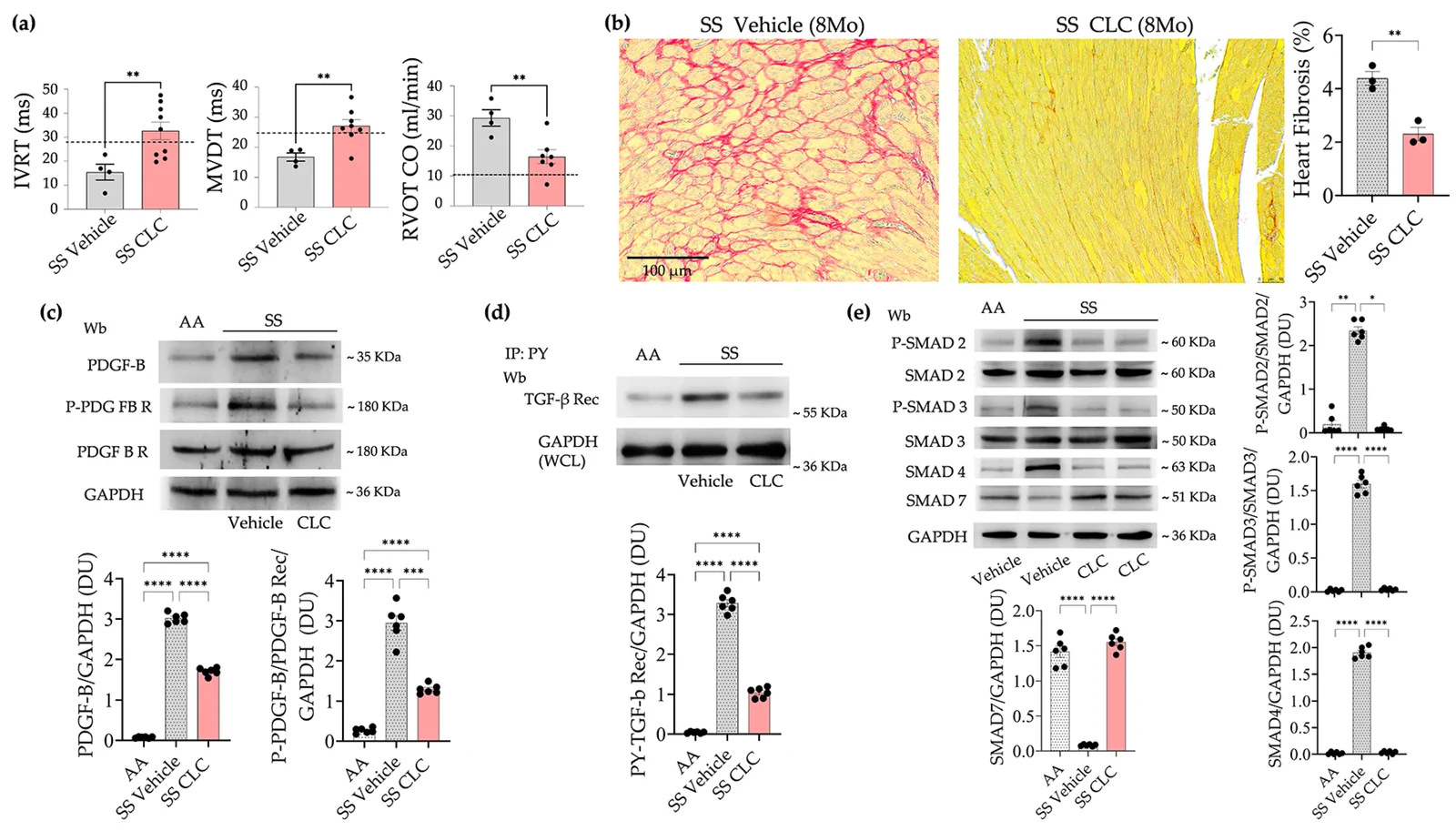
**In sickle cell mice, CLC protects against age-dependent heart fibrosis, preventing the activation of pro-fibrotic pathways.** (**a**) Isovolumetric relaxation time (IVRT), mitral valve deceleration time (MVDT), and right ventricular cardiac output (RVCO) assessed by echocardiography in SS mice treated with either vehicle or CLC. Dashed lines indicate the corresponding values observed in AA mice, as we previously reported [4]. (**b**) Picrosirius Red staining in cardiac slices from SS mice treated with either vehicle or CLC. **Right panel**: Quantification of heart fibrosis. (**c**) Immunoblot analysis, using specific antibodies against PDGFB, phosphorylated p-PDGFR-B and PDGFR-B in heart from healthy (AA) and SS mice treated as in a. 75 µg/µL of protein loaded on an 8% T, 2.5%C polyacrylamide gel. GAPDH serves as protein loading control. One representative gel from 5 with similar results is shown. Densitometric analysis of immunoblots is shown on the bottom. (**d**) Immunoprecipitation from heart healthy (AA) and SS mice treated as in a, using specific anti-phospho-Tyrosine antibodies (IP: PY), revealed with specific anti-TGF β receptor (Rec) antibody (75 µg/µL of protein loaded on an 8% T, 2.5%C polyacrylamide gel). GAPDH in whole cell lysate (WCL) is used as loading controls. Densitometric analysis of immunoblots is shown on the bottom. (**e**) Immunoblot analysis using specific antibodies against phosphorylated (p-) Smad 2, Smad 2, p-Smad3, Smad 3, Smad 4 and Smad 7 in heart from in heart from healthy (AA) and SS mice treated as in a. One representative gel from 6 gels with similar results is shown. 75 µg/µL of protein loaded on an 11% T, 2.5%C polyacrylamide gel. GAPDH serves as protein loading control. Densitometric analysis of immunoblots is shown in the right and bottom panels. Data in the column charts are presented as individual data points with mean ± SEM. Significance codes: * *p* < 0.05; ** *p* < 0.01; *** *p* < 0.001; **** *p* < 0.0001; not significant comparisons were not reported. Adjusted *p*-values were used when multiple comparisons were involved.

In different models of cardiovascular diseases, CLC has been described to act on multiple targets going from activation of NF-kB pathways and NLRP3 inflammasome to inhibition of TGF- b system [18,36,37,38,39]. Since mild chronic inflammation and overactivation of profibrotic pathways together with local immune cell recruitment characterizes the development of sickle cell related cardiomyopathy, we decided to investigate the impact of CLC on the natural history of sickle cell related cardiomyopathy. In SS mice, CLC was well tolerated. No change in mouse weight, no diarrhea, no change in liver function was observed in CLC treated SS mice compared to vehicle treated animals (Supplementary Figure S5a,b). CLC treatment prevented the diastolic dysfunction previously characterized in SS mice [4]. Indeed, echocardiographic analysis demonstrated improvement of established diastolic function indices, including isovolumetric relaxation time (IVRT) and mitral valve deceleration time (MVDT), as well as restoration of right ventricular cardiac output (RVCO) to levels comparable to those observed in AA mice (Figure 4a; Supplementary Table S1).

In agreement with the functional recovery, CLC treated 8-months old SS mice displayed a significant reduction in collagen heart deposition when compared to vehicle treated animals (Figure 4b). This was associated with the reduction in PDFGF-B expression and in the activation of pro-fibrotic signaling pathways involving PDFG-B and FGF receptors (Figure 4c). As expected, we found reduced activation of TGF-b receptor and of its canonical pathway involving SMADS system (Figure 4d,e). We compared the effects of CLC on sickle cell cardiomyopathy with those of HU. In HU treated animals, we observed a significant increase in Hb when compared to vehicle treated SS mice (Supplementary Figure S6a). HU treated sickle cell mice displayed downregulation of PDGF-B expression associated with slight reduction in PDGF-B receptor activation and persistent activation of FGF-receptor when compared to vehicle treated animals (Supplementary Figure S6b). We also observed a slight but not significant reduction in TGF-b receptor activation in HU treated SS mice compared to vehicle treated animals (Supplementary Figure S6c). Collectively, our data support the protective effect of CLC, and its superiority compared to HU treatment against the age dependent progression of heart fibrosis in SCD mice.

### 3.4. In SS Mice, CLC Attenuates Inflammatory Cell Infiltrates and Positively Modulates the Local NF-kB Related Pro-Inflammatory Response

**Figure 5:**
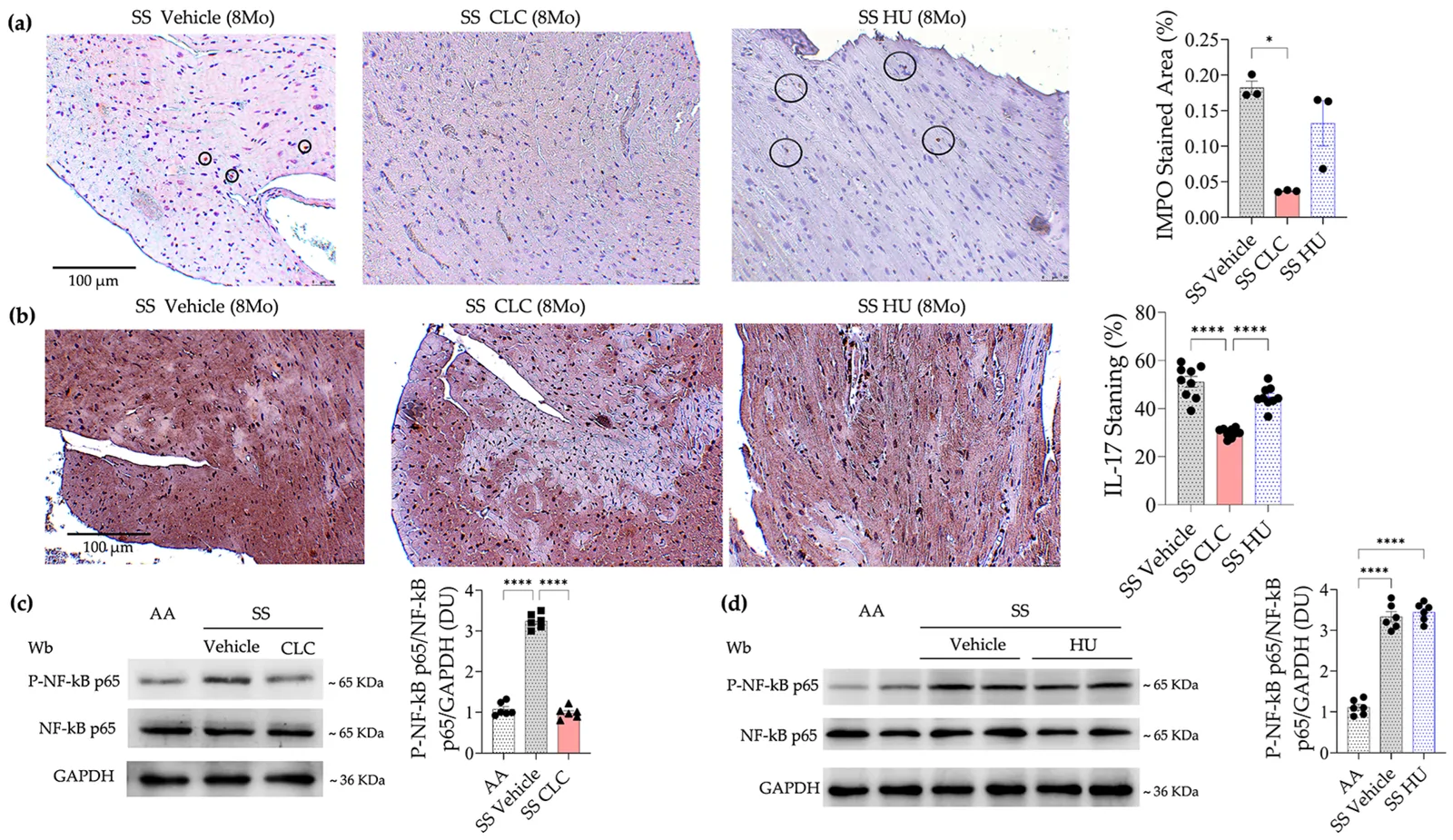
**CLC but not hydroxyurea attenuates heart inflammatory cell recruitment and prevents the activation of heart NF-kB.** (**a**) Representative myeloperoxidase (MPO) immunohistochemical staining of cardiac slices from SS mice treated with either vehicle, CLC or hydroxyurea (HU). MPO quantification is shown on the right. Data are presented as mean ± SEM (n = 3). ** *p* < 0.005 by *t*-test. Circles highlight MPO-positive cells. (**b**) Representative IL-17 immunohistochemical staining in cardiac slices from SS mice treated with either vehicle, CLC or hydroxyurea (HU). IL-17 quantification is shown on the right. (**c**) Immunoblot analysis, using specific antibodies against phosphorylated (p)NF-κB p65 and NF-κB p65, of heart from healthy (AA) and SS mice treated as in a. 75 µg/µL of protein loaded on an 8% T, 2.5%C polyacrylamide gel. GAPDH serves as protein loading control. One representative gel from 6 with similar results is shown. Densitometric analysis of immunoblots is shown on the right. (**d**) Immunoblot analysis, using specific antibodies against phosphorylated (p)NF-κB p65 and NF-κB p65, of heart from healthy (AA) and SS mice treated with either vehicle or HU. 75 µg/µL of protein loaded on an 8% T, 2.5%C polyacrylamide gel. GAPDH serves as protein loading control. One representative gel from 6 with similar results is shown. Densitometric analysis of immunoblots is shown on the right. Data in the column charts are presented as individual data points with mean ± SEM. Significance codes: * *p* < 0.05; ** *p* < 0.01; *** *p* < 0.001; **** *p* < 0.0001; not significant comparisons were not reported. Adjusted *p*-values were used when multiple comparisons were involved.

In 8-month-old SS, CLC significantly reduced heart neutrophil infiltration and IL17A heart staining when compared to either vehicle or HU treated animals (Figure 5a,b).

**Figure 6:**
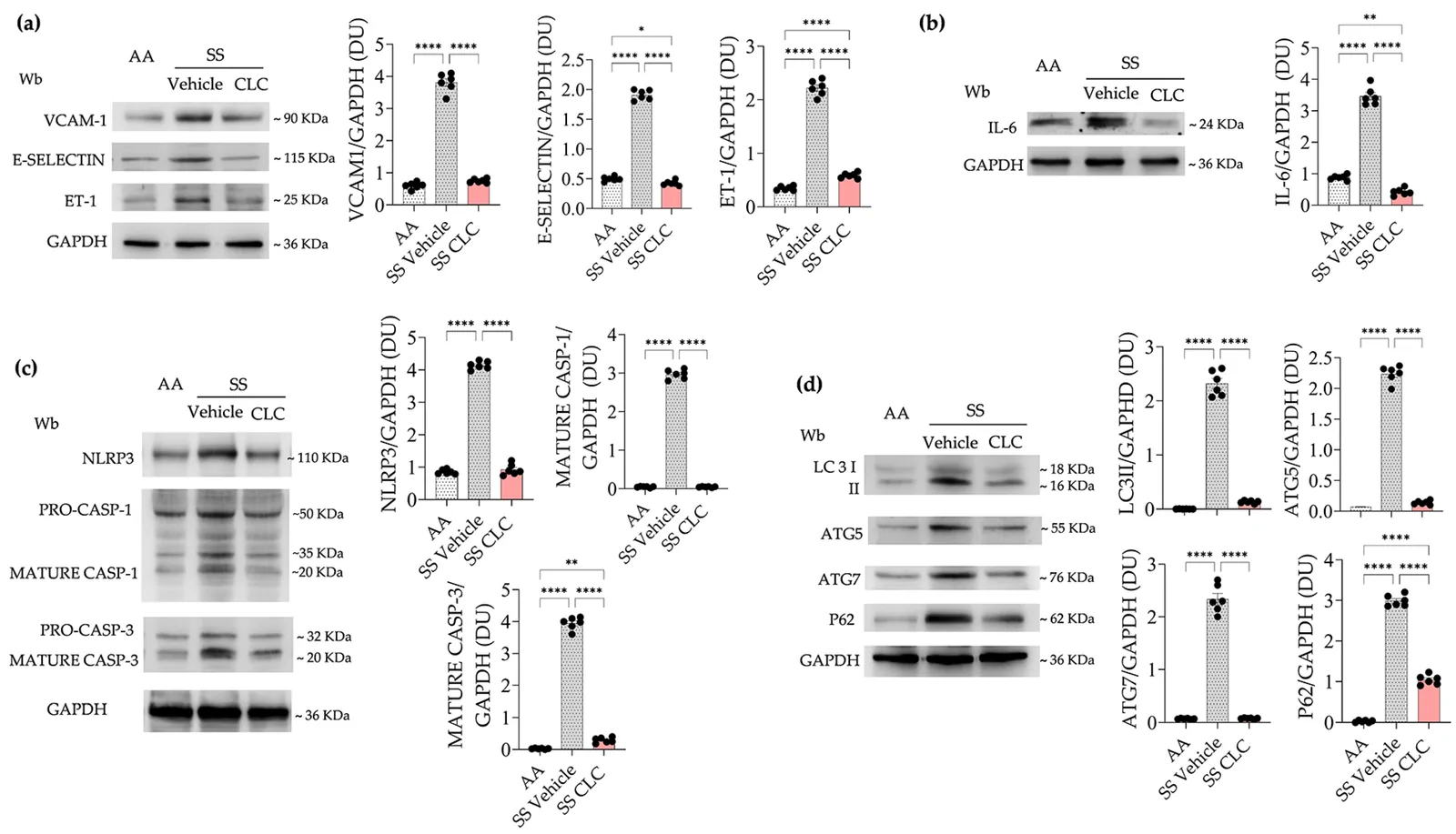
**CLC protects against inflammatory vasculopathy and the activation of NLRP3 inflammasome, resulting in improved autophagy.** (**a**) Immunoblot analysis, using specific antibodies against vascular cell adhesion molecule-1 (VCAM-1), E Selectin and endothelin-1 (ET-1) in heart from AA and SS mice treated with either vehicle or CLC. 75 µg/µL of protein loaded on an 11% T, 2.5%C polyacrylamide gel. Actin serves as protein loading control. One representative gel from 6 with similar results is shown. Densitometric analysis of immunoblots is shown on the right. (**b**) Immunoblot analysis, using specific antibodies against IL-6 in heart from AA and SS mice treated with either vehicle or CLC. 75 µg/µL of protein loaded on an 11% T, 2.5%C polyacrylamide gel. Actin serves as protein loading control. One representative gel from 6 with similar results is shown. Densitometric analysis of immunoblots is shown on the right. (**c**) Immunoblot analysis, using specific antibodies against NLRP3, Caspase 1 (CASP1) and Caspase 3 (CASP3) in heart from AA and SS mice treated as in a. 75 µg/µL of protein loaded on an 8% T, 2.5%C polyacrylamide gel. GAPDH serves as protein loading control. One representative gel from 6 with similar results is shown. Densitometric analysis of immunoblots is shown in the right panel. (**d**) Immunoblot analysis, using specific antibodies against LC3I/II, ATG5, 7 and P62 in heart from AA and SS mice treated as in a. 75 µg/µL of protein loaded on an 8% T, 2.5%C polyacrylamide gel. GAPDH serves as protein loading control. One representative gel from 6 with similar results is shown. Densitometric analysis of immunoblots is shown in the right panel. Data in the column charts are presented as individual data points with mean ± SEM. Significance codes: * *p* < 0.05; ** *p* < 0.01; *** *p* < 0.001; **** *p* < 0.0001; not significant comparisons were not reported. Adjusted *p*-values were used when multiple comparisons were involved.

This was associated with a significant reduction in plasma IL17 and in heart recruited Th17 cells (Supplementary Figure S7a,b). Notably, CLC also reduced peripheral and spleen Th17 when compared to vehicle treated animals (Supplementary Figure S7c). Similarly, in CLC treated SS mice, we found a significant reduction in the activation of heart NF-kB, whereas no major change in NF-kB activation was observed in heart from HU treated SS mice when compared to vehicle treated animals (Figure 5c,d). Indeed, in heart from SS mice, CLC significantly down-regulated the expression of inflammatory vasculopathy (VCAM-1, E-Selectin, ET-1) and of IL-6 when compared to vehicle treated mice (Figure 6a,b).

This was associated with reduced activation of NLRP3 inflammasome and its related pro-apoptotic pathway involving caspase-1 and caspase-3 (Figure 6c). We found a significant reduction in the expression of both mature caspase-1 and -3 in heart from CLC treated mice when compared to vehicle treated animals. Of note, no change in heart NLRP3 activation was observed in SS mice treated with HU when compared to vehicle treated animals (Supplementary Figure S8a). We then investigated whether CLC’s cardioprotective effects might be mediated by the activation of molecular pro-resolving circuits. Building on our recent discovery of a miRNA module regulated by 17R-resolvin D1 (17R-RvD1) in SCD cardiomyopathy, we assessed the expression of NF-KB related miRNA targets in CLC-treated SS mice. CLC treatment significantly downregulated miR-16, miR-25, and miR-208-3p (Supplementary Figure S8b). Given their established roles in cardiac fibrosis, arrhythmia, and cardiomegaly, these data suggest that CLC confers protection by activating miRNA-driven pro-resolving pathways in the heart from SCD mice. Previous studies have shown a link between the activation of NLRP3 and impaired autophagy promoting cardiomyocyte disfunction [40,41]. In CLC treatment reduced LC3II and prevented the accumulation of autophagy related proteins such as ATG5 and 7 as well as p62, involving the terminal phase of autophagy when compared to vehicle treated animals (Figure 6d).

Collectively, our data indicate that CLC reduced systemic and local inflammation, prevented aging induced activation of heart NF-kB pro-inflammatory pathways and NLRP3, associated with an improvement of autophagy.

Since heart and lung systems are functionally interconnected [42] and lung is one of the target organs of SCD, we asked whether CLC treatment might be protective against sickle cell related lung disease. This is also supported by previous studies showing that CLC attenuates acute and chronic lung damage [43,44].

### 3.5. In SCD Mice, CLC Has Beneficial Effects Beyond the Heart, Resulting in Protection of the Lung and, Potentially, Systemic Inflammatory Vasculopathy

**Figure 7:**
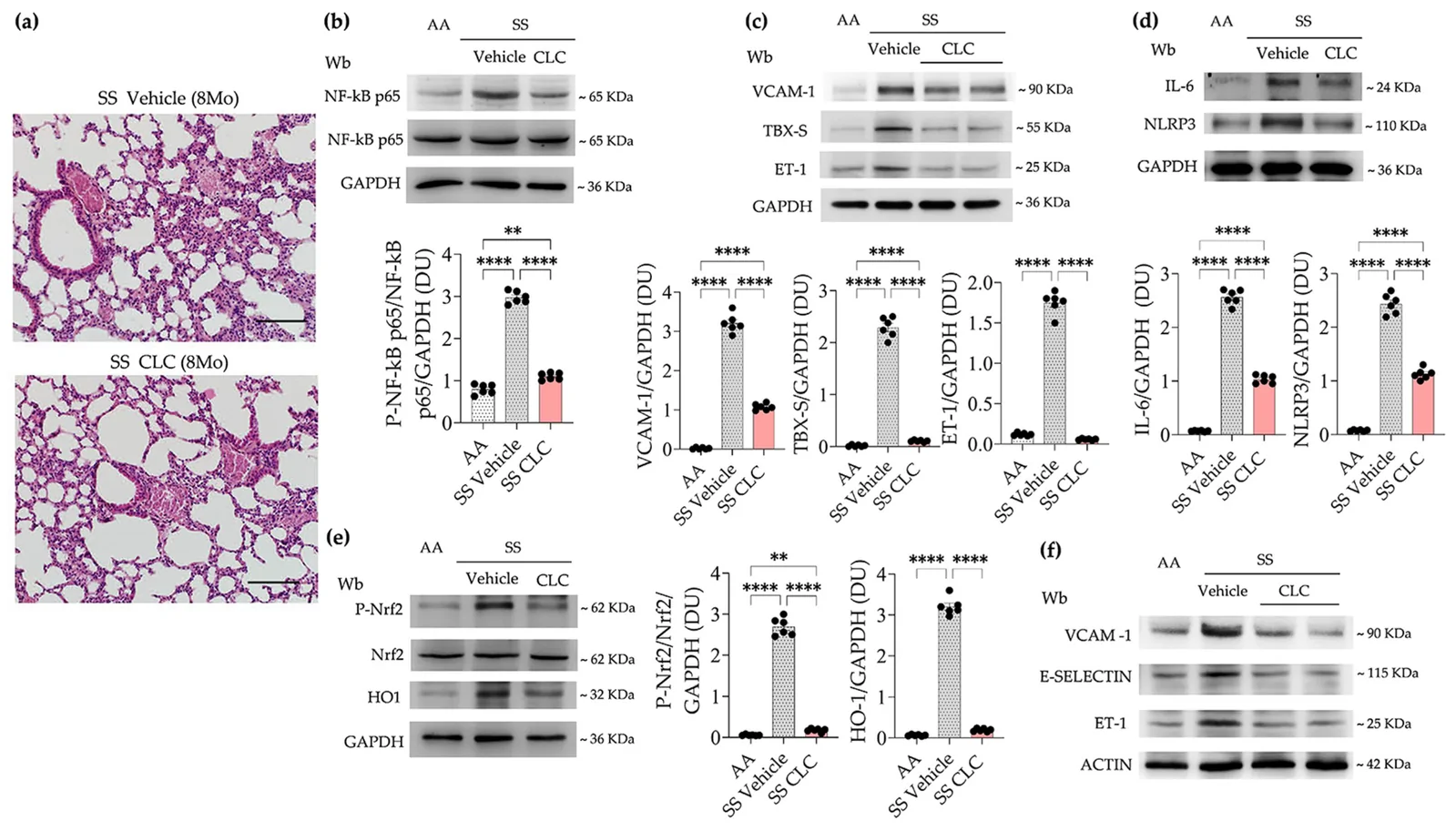
**CLC reduces lung inflammatory cell infiltrates, preventing the activation of NF-KB and NRF2, and protects against inflammatory vasculopathy in lung and isolated aorta.** (**a**) Representative micro-picture of hematoxylin and eosin-stained sections at 200x magnification form sickle cell mice at 3 and 8 months of age. (**b**) Immunoblot analysis, using specific antibodies against phosphorylated (p)NF-κB p65 and NF-κB p65, of lung from healthy (AA) and SS mice treated with either vehicle or HU. 75 µg/µL of protein loaded on an 8% T, 2.5%C polyacrylamide gel. GAPDH serves as protein loading control. One representative gel from 6 with similar results is shown. Densitometric analysis of immunoblots is shown on the right. (**c**) Immunoblot analysis, using specific antibodies against vascular cell adhesion molecule-1 (VCAM-1), Thromboxane synthase -1 (TBXS) and endothelin-1 (ET-1) in lung from AA and SS mice treated with either vehicle or CLC. 75 µg/µL of protein loaded on an 11% T, 2.5%C polyacrylamide gel. GAPDH serves as protein loading control. One representative gel from 6 with similar results is shown. Densitometric analysis of immunoblots is shown in the bottom panel. (**d**) Immunoblot analysis, using specific antibodies against IL-6 and NLRP3 in lung from AA and SS mice treated with either vehicle or CLC. 75 µg/µL of protein loaded on an 11% T, 2.5%C polyacrylamide gel. GAPDH serves as protein loading control. One representative gel from 6 with similar results is shown. Densitometric analysis of immunoblots is shown on the right. (**e**) Immunoblot analysis, using specific antibodies against phosphor (p)-NRF2, NRF2 and HO-1 in lung from AA and SS mice treated with either vehicle or CLC. 75 µg/µL of protein loaded on an 11% T, 2.5%C polyacrylamide gel. GAPDH serves as protein loading control. One representative gel from 6 with similar results is shown. Densitometric analysis of immunoblots is shown on the right. (**f**) Immunoblot analysis, using specific antibodies against vascular cell adhesion molecule-1 (VCAM-1), E-Selectin and endothelin-1 (ET-1) in isolated aorta from AA and SS mice treated with either vehicle or CLC. 75 µg/µL of protein loaded on an 11% T, 2.5%C polyacrylamide gel. Actin serves as protein loading control. One representative gel from 6 with similar results is shown. Densitometric analysis of immunoblots is shown Supplementary Figure S9. Data in the column charts are presented as individual data points with mean ± SEM. Significance codes: ** *p* < 0.01; *** *p* < 0.001; **** *p* < 0.0001; not significant comparisons were not reported. Adjusted *p*-values were used when multiple comparisons were involved.

As shown in Figure 7a, CLC treated SCD mice displayed lower inflammatory cell infiltrates than vehicle treated animals.

This was associated with reduction in the activation of NF-kB (Figure 7b). In agreement, we observed down-regulation of (i) markers of inflammatory vasculopathy such as VCAM-1, TBX1 and ET1; (ii) IL-6 and NLRP3 inflammasome (Figure 7c,d). We also found reduced activation of Nrf2, a transcriptional factor modulating the expression of antioxidant and cytoprotective systems (Figure 7e). In agreement, we found down-regulation of HO-1 expression (Figure 7e). Given the beneficial effects of CLC in models of inflammatory vasculopathy such as atherosclerosis, we tested the impact of CLC treatment in 8-months old mice. As shown in Figure 7f, in 8-months old SCD mice, CLC down-regulated the aorta expression of markers of inflammatory vasculopathy such as VCAM-1, E-selectin and ET-1 when compared to vehicle treated SS animals and reaching values similar to those observed in aged matched healthy mice (Supplementary Figure S9). Taken together, our data indicate that the beneficial effect of CLC might go beyond heart, resulting a protection of the axis between lung and heart, protecting against systemic inflammatory vasculopathy.

## 4. Discussion

Here, we studied the natural history of sickle cell related cardiomyopathy in humanized SS mice. We found that mild chronic inflammation or unresolved inflammation plays a key role in heart disease progression towards cardiac fibrosis and myocardial dysfunction. In young SS mice, we previously showed that H/R stress initiates a cardiac-hypertrophic neutrophil-driven response [4]. In aging SS mice under normoxia, the persistent mild chronic inflammation promotes neutrophil and Th17 heart infiltration associated with heart overactivation of NF-kB dependent pro-inflammatory and pro-fibrotic pathways. This generates a premature senescence environment, which is considered a marker inflammaging. Inflammaging has been recently proposed as cumulative risk factor for cardiovascular disease (CVD) even in young adults affected by clinical conditions characterized by mild chronic inflammation such as the metabolic syndrome [45,46]. Inflammaging is characterized by a peculiar signaling spectrum, named senescence-associated secretory phenotype (SASP), which involves cytokines and chemokines as IL17, IL6 and CCL2 [47,48]. In aging SS mice, we found increased soluble IL-17 and CCL2 as well as heart expression of IL-17 and IL-6. In SS mice, these (i) contribute to heart inflammatory cell infiltrates by neutrophil and Th17; (ii) promote inflammatory vasculopathy as supported by increase expression of VCAM-1 and selectins; and (iii) pro-fibrotic signaling involving ET-1 and TGF-β canonical pathway. Of note, we found an age-dependent increase in heart neutrophil recruitment, whereas heart Th17 infiltration was detectable in both young and old SS mice. This was paralleled by age-dependent increased in both circulating and spleen Th17 when compared to healthy mice, contributing to the senescent immunophenotype of SS mice [49].

Recent studies have highlighted the importance of lymphocytes in pathologic heart remodeling and in heart disease progression [45,50]. Here, we newly identified Th17 heart recruitment together with age-dependent neutrophil heart infiltration in aging SCD mice. Notably, in SS mice, the increased IL17 might play a pivotal role in the cooperation between neutrophil and Th17 towards heart fibrosis also throughout the modulation of CCL2 [32,34,51] This is also supported by studies in different models of CVD, showing that IL17 polymorphism is linked with severe cardiovascular disease and that IL17 deletion is protective against H/R stress in rodents. [51,52]. In addition, IL17 might up-regulate the expression of both ET-1 and VCAM-1, contributing to inflammatory vasculopathy [52]. Indeed, SCD mice displayed increased expression of markers of inflammatory vasculopathy when compared to healthy animals. Our data, indicate that in SS mice inflammaging might exacerbate myocardial disfunction by heart fibrosis involved in SS related cardiomyopathy. Previous studies highlight the novel role of CLC as anti-inflammatory drug with immunomodulatory effects against inflammatory vasculopathy and CVD [18,39,53]. This is based on the multimodal action of CLC by (i) inhibition of microtubule polymerization that is important in NF-kB activation and NLRP3 activation; (ii) the down-regulation of CRP as well as of pro-inflammatory cytokines/chemokines; (iii) the modulation of adaptive innate immunity and neutrophil/lymphocytes organ recruitment; (iv) the decrease of TGF-b mediated pro-fibrotic canonical pathway; and (v) the down-regulation of miR-16, miR-25, and miR-208-3p, involved in cardiac fibrosis, arrhythmia, and cardiomegaly [4,37,38,39,53,54]. Thus, CLC might be considered a therapeutic option against inflammaging in CVD. Notably, Fouda et al. recently reported that in “Berk” SCD mice short term (2 weeks) treatment with CLC reduced systemic inflammation and decreased skin mast cell degranulation when compared to vehicle treated animals in the absence of change in soluble IL6, IL17 or MCP1 [55]. This might be related to the short period of treatment and to the different dosage of CLC used [55].

Here, we found that CLC protected SS mouse heart performance, prevented the activation of NF-kB mediated pro-inflammatory and prof-fibrotic pathways. CLC showed superiority against cardiac fibrosis via TGF-b pathway and local inflammation by NF-kB and NLRP3 activation and increased heart IL17 staining when compared to HU. Whereas CLC and HU similarly reduced PDGF-B heart expression, supporting the reported protective effect of HU. This is mainly indirect due to improvement of anemia and the reported beneficial effects on inflammatory vasculopathy. Otherwise, in SS mice CLC has direct effects on systemic and heart chronic inflammation without improvement of the chronic anemia. This is also supported by the improvement of heart autophagy, which contributes to prevent the activation of pro-apoptotic cellular profile in CLC treated SS mice when compared to vehicle treated animals. Our observation agrees with the reported protective effect of low dose CLC by restoring the late phase autophagy against doxorubicin-induced cardiotoxicity [56]. CLC has been reported to display a lung protective effect in both acute and chronic settings [43,44,57]. In lung from 8 month old mice SS mice, CLC (i) reduced the lung inflammatory cell infiltrates; (ii) prevented the activation of NF-kB and NLRP3 inflammasome; (ii) down-regulated the lung expression of markers of inflammatory vasculopathy and of local inflammation; (iii) prevented the activation of Nrf2, a transcription factor related to oxidative response; and (iv) decreased the lung expression of HO-1. Collectively, our data indicates that CLC protects against chronic lung inflammation and attenuating the inflammatory vasculopathy.

Although our study shed new lights on the natural history of sickle cell-related cardiomyopathy, we identified some study limitations such as the relatively small sample sizes used in some of the histologic measurements; the use of surrogate markers (e.g., MPO) for inflammatory heart cell infiltration and for inflammasome activity (e.g., pro-caspase 1/mature caspase 1). These might be addressed in future studies considering also the combo therapy CLC and HU and the effects of CLC on other target organs for sickle cell disease such as liver or kidney.

## 5. Conclusions

In conclusion, we show for the first time the role of inflammaging in the natural history of sickle cell related cardiomyopathy. We identified the contribution of IL17 and Th17 in progression of cardiac fibrosis involving the activation of TGF-b canonical pathway. We demonstrated the protective role of CLC against chronic inflammation, pathologic heart remodeling, and preserving myocardial performance. Since CLC is safe and generally well tolerated at low dosage, we suggest to possibly consider CLC for early treatment of SS patients with diastolic dysfunction, a functional marker of cardiac fibrosis. However, the current evidence on clinical management of different cardiovascular diseases with CLC is still limited and mainly coming from pre-clinical studies [18]. Thus, the translational of CLC treatment in clinical management of patients with SCD still requires validation in future clinical studies.

## Data Availability

All data are stored in the Nas-Synology-DS216se-Hard-Disk, located at the University of Verona and will be available on request.

## Supplementary Materials

The following supporting information can be downloaded at: https://www.mdpi.com/article/10.3390/antiox15080912/s1, Table S1. Echocardiographic parameters of sickle cell (SS) mice treated with either vehicle or colchicine (CLC); Figure S1. Hemoglobin, cardiomyocyte area and heart SERCA2A expression aging sickle cell mice; Figure S2. Densitometric analysis of Figure 2a; Figure S3. Quantification of heart IL17; Figure S4. Gating strategy and quantification of peripheral and splenic Th17 lymphocytes; Figure S5. Mouse weight and liver enzymes in sickle cell mice treated with either vehicle or colchicine; Figure S6. Hemoglobin, heart expression and degree of activation of PDGF-B/FGF-receptors; Figure S7. Plasma IL17A, heart Th17 lymphocytes infiltration, peripheral and splenic Th17 lymphocytes. Figure S8. Heart expression of NLRP3 and pro-resolving miRNAs; Figure S9. Densitometric analysis of immunoblots in Figure 7f.

## Abbreviations

The following abbreviations are used in this manuscript:

SCD	Sickle cell disease
VOC	Vaso Occlusive Crisis
CLC	Colchicine
HU	Hydroxyurea
Hb	Hemoglobin
SS mice	Sickle cell mice
SERCA2A	Sarcoplasmic reticulum calcium ATPase cardiac isoform
CVD	Cardiovascular Disease
AST	Aspartate aminotransferase
ALT	Alanine aminotransferase
NF-kB	Nuclear Factor kappa-light-chain-enhancer of activated B cells
TGF-β	Transforming Growth Factor Beta
PDGF-B	Platelet-Derived Growth Factor Subunit B
FGF	Fibroblast Growth Factor
ET-1	Endothelin 1
CCL2	C-C Motif Chemokine Ligand 2
IL17	Interleukin 17
NLRP3	Nucleotide-binding domain, Leucine-rich repeat, and Pyrin domain-containing protein 3
VCAM-1	Vascular Cell Adhesion Molecule-1
IL-6	Interleukin 6
17R-RvD1	17R-resolvin D1
ATG 5	Autophagy protein 5
ATG 7	Autophagy protein 7
HO-1	Heme oxygenase-1
TBX1	T-box transcription factor 1

## References

[1] Sachdev V., Machado R.F., Shizukuda Y., Rao Y.N., Sidenko S., Ernst I., St Peter M., Coles W.A., Rosing D.R., Blackwelder W.C. (2007). Diastolic dysfunction is an independent risk factor for death in patients with sickle cell disease. J. Am. Coll. Cardiol..

[2] Sachdev V., Rosing D.R., Thein S.L. (2021). Cardiovascular complications of sickle cell disease. Trends Cardiovasc. Med..

[3] Matte A., Recchiuti A., Federti E., Koehl B., Mintz T., El Nemer W., Tharaux P.L., Brousse V., Andolfo I., Lamolinara A. (2019). Resolution of sickle cell disease-associated inflammation and tissue damage with 17R-resolvin D1. Blood.

[4] Federti E., Mattoscio D., Recchiuti A., Matte A., Monti M., Cozzolino F., Iezzi M., Ceci M., Ghigo A., Tolosano E. (2025). 17(R)-Resolvin D1 Protects against Sickle Cell-Related Inflammatory Cardiomyopathy in Humanized Mice. Blood.

[5] Bakeer N., James J., Roy S., Wansapura J., Shanmukhappa S.K., Lorenz J.N., Osinska H., Backer K., Huby A.C., Shrestha A. (2016). Sickle cell anemia mice develop a unique cardiomyopathy with restrictive physiology. Proc. Natl. Acad. Sci. USA.

[6] Gupta A., Fei Y.D., Kim T.Y., Xie A., Batai K., Greener I., Tang H., Ciftci-Yilmaz S., Juneman E., Indik J.H. (2021). IL-18 mediates sickle cell cardiomyopathy and ventricular arrhythmias. Blood.

[7] Wagdy R., Assem H., Abd-Elmohsen A.M., Fata A., Gendy W.E., Gaber M. (2024). Altered ventricular longitudinal strain in children with sickle cell disease: Role of TGF-beta and IL-18. Pediatr. Blood Cancer.

[8] Niss O., Detterich J., Wood J.C., Coates T.D., Malik P., Taylor M.D., Quinn C.T. (2022). Early initiation of disease-modifying therapy can impede or prevent diffuse myocardial fibrosis in sickle cell anemia. Blood.

[9] Thomas T.P., Grisanti L.A. (2020). The Dynamic Interplay Between Cardiac Inflammation and Fibrosis. Front. Physiol..

[10] Yang K., Liu Q., Fan A., Lin H., Wang X., Cui T., Fan G., Li L. (2025). Th17 Cells in Cardiovascular Disease. Cell Biochem. Funct..

[11] Ma L., Yang W., Gao W., Liu X., Dong M., An G., Meng X. (2025). IL-17 as a therapeutic target in cardiovascular diseases: Mechanistic insights and translational opportunities. Pharmacol. Res..

[12] Chen G., Bracamonte-Baran W., Diny N.L., Hou X., Talor M.V., Fu K., Liu Y., Davogustto G., Vasquez H., Taegtmeyer H. (2018). Sca-1(+) cardiac fibroblasts promote development of heart failure. Eur. J. Immunol..

[13] Garcia N.P., Junior A.L.S., Soares G.A.S., Costa T.C.C., Dos Santos A.P.C., Costa A.G., Tarrago A.M., Martins R.N., do Carmo Leao Pontes F., de Almeida E.G. (2020). Sickle Cell Anemia Patients Display an Intricate Cellular and Serum Biomarker Network Highlighted by TCD4+CD69+ Lymphocytes, IL-17/MIP-1beta, IL-12/VEGF, and IL-10/IP-10 Axis. J. Immunol. Res..

[14] Allali S., Dietrich C., Machavoine F., Rignault-Bricard R., Brousse V., de Montalembert M., Hermine O., Maciel T.T., Leite-de-Moraes M. (2019). Innate-like T cells in children with sickle cell disease. PLoS ONE.

[15] Marchesani S., Bertaina V., Marini O., Cossutta M., Di Mauro M., Rotulo G.A., Palma P., Sabatini L., Petrone M.I., Frati G. (2022). Inflammatory status in pediatric sickle cell disease: Unravelling the role of immune cell subsets. Front. Mol. Biosci..

[16] Deftereos S.G., Beerkens F.J., Shah B., Giannopoulos G., Vrachatis D.A., Giotaki S.G., Siasos G., Nicolas J., Arnott C., Patel S. (2022). Colchicine in Cardiovascular Disease: In-Depth Review. Circulation.

[17] Trube J., Sabina M., Khanani A., Hernandez K., Khan Z., Bizanti A. (2025). Colchicine therapy in cardiovascular medicine: A literature review. Am. Heart J. Plus.

[18] Buckley L.F., Libby P. (2024). Colchicine’s Role in Cardiovascular Disease Management. Arterioscler. Thromb. Vasc. Biol..

[19] Imazio M., Brucato A., Trinchero R., Spodick D., Adler Y. (2009). Colchicine for pericarditis: Hype or hope?. Eur. Heart J..

[20] De Franceschi L., Bachir D., Galacteros F., Tchernia G., Cynober T., Alper S., Platt O., Beuzard Y., Brugnara C. (1997). Oral magnesium supplements reduce erythrocyte dehydration in patients with sickle cell disease. J. Clin. Investig..

[21] Fujisue K., Sugamura K., Kurokawa H., Matsubara J., Ishii M., Izumiya Y., Kaikita K., Sugiyama S. (2017). Colchicine Improves Survival, Left Ventricular Remodeling, and Chronic Cardiac Function After Acute Myocardial Infarction. Circ. J..

[22] Chia E.W., Grainger R., Harper J.L. (2008). Colchicine suppresses neutrophil superoxide production in a murine model of gouty arthritis: A rationale for use of low-dose colchicine. Br. J. Pharmacol..

[23] McLoughlin E.C., O’Boyle N.M. (2020). Colchicine-Binding Site Inhibitors from Chemistry to Clinic: A Review. Pharmaceuticals.

[24] Slobodnick A., Shah B., Pillinger M.H., Krasnokutsky S. (2015). Colchicine: Old and new. Am. J. Med..

[25] de Franceschi L., Rouyer-Fessard P., Alper S.L., Jouault H., Brugnara C., Beuzard Y. (1996). Combination therapy of erythropoietin, hydroxyurea, and clotrimazole in a beta thalassemic mouse: A model for human therapy. Blood.

[26] Matte A., Federti E., Recchiuti A., Hamza M., Ferri G., Riccardi V., Ceolan J., Passarini A., Mazzi F., Siciliano A. (2024). Epeleuton, a novel synthetic omega-3 fatty acid, reduces hypoxia/ reperfusion stress in a mouse model of sickle cell disease. Haematologica.

[27] Brugnara C., de Franceschi L. (1993). Effect of cell age and phenylhydrazine on the cation transport properties of rabbit erythrocytes. J. Cell. Physiol..

[28] Li M., Sala V., De Santis M.C., Cimino J., Cappello P., Pianca N., Di Bona A., Margaria J.P., Martini M., Lazzarini E. (2018). Phosphoinositide 3-Kinase Gamma Inhibition Protects From Anthracycline Cardiotoxicity and Reduces Tumor Growth. Circulation.

[29] Schnelle M., Catibog N., Zhang M., Nabeebaccus A.A., Anderson G., Richards D.A., Sawyer G., Zhang X., Toischer K., Hasenfuss G. (2018). Echocardiographic evaluation of diastolic function in mouse models of heart disease. J. Mol. Cell. Cardiol..

[30] Aldo P., Marusov G., Svancara D., David J., Mor G. (2016). Simple Plex(): A Novel Multi-Analyte, Automated Microfluidic Immunoassay Platform for the Detection of Human and Mouse Cytokines and Chemokines. Am. J. Reprod. Immunol..

[31] Federti E., Vinchi F., Iatcenko I., Ghigo A., Matte A., Toya S.C.M., Siciliano A., Chiabrando D., Tolosano E., Vance S.Z. (2023). Duality of Nrf2 in iron-overload cardiomyopathy. Haematologica.

[32] Georgakis M.K., de Lemos J.A., Ayers C., Wang B., Bjorkbacka H., Pana T.A., Thorand B., Sun C., Fani L., Malik R. (2021). Association of Circulating Monocyte Chemoattractant Protein-1 Levels With Cardiovascular Mortality: A Meta-analysis of Population-Based Studies. JAMA Cardiol..

[33] Georgakis M.K., Malik R., Bjorkbacka H., Pana T.A., Demissie S., Ayers C., Elhadad M.A., Fornage M., Beiser A.S., Benjamin E.J. (2019). Circulating Monocyte Chemoattractant Protein-1 and Risk of Stroke: Meta-Analysis of Population-Based Studies Involving 17 180 Individuals. Circ. Res..

[34] Du S., Li Z., Xie X., Xu C., Shen X., Wang N., Shen Y. (2020). IL-17 stimulates the expression of CCL2 in cardiac myocytes via Act1/TRAF6/p38MAPK-dependent AP-1 activation. Scand. J. Immunol..

[35] Santos-Zas I., Lemarie J., Tedgui A., Ait-Oufella H. (2018). Adaptive Immune Responses Contribute to Post-ischemic Cardiac Remodeling. Front. Cardiovasc. Med..

[36] Suryono S., Rohman M.S., Widjajanto E., Prayitnaningsih S., Wihastuti T.A. (2023). Colchicine as potential inhibitor targeting MMP-9, NOX2 and TGF-beta1 in myocardial infarction: A combination of docking and molecular dynamic simulation study. J. Biomol. Struct. Dyn..

[37] Yue H., Liang W., Zhan Y., Zhang Z., Qin X., Bian L., He K., Wu Z. (2022). Colchicine: Emerging therapeutic effects on atrial fibrillation by alleviating myocardial fibrosis in a rat model. Biomed. Pharmacother..

[38] Shen S., Duan J., Hu J., Qi Y., Kang L., Wang K., Chen J., Wu X., Xu B., Gu R. (2022). Colchicine alleviates inflammation and improves diastolic dysfunction in heart failure rats with preserved ejection fraction. Eur. J. Pharmacol..

[39] Akodad M., Sicard P., Fauconnier J., Roubille F. (2020). Colchicine and myocardial infarction: A review. Arch. Cardiovasc. Dis..

[40] Mauro A.G., Mezzaroma E., Toldo S., Melendez G.C., Franco R.L., Lesnefsky E.J., Abbate A., Hundley W.G., Salloum F.N. (2023). NLRP3-mediated inflammation in cardio-oncology: Sterile yet harmful. Transl. Res..

[41] Lv S., Wang H., Li X. (2021). The Role of the Interplay Between Autophagy and NLRP3 Inflammasome in Metabolic Disorders. Front. Cell Dev. Biol..

[42] Ahmad S., Isbatan A., Chen S., Dudek S.M., Minshall R.D., Chen J. (2024). The Interplay of Heart Failure and Lung Disease: Clinical Correlations, Mechanisms, and Therapeutic Implications. J. Respir. Biol. Transl. Med..

[43] Li F., Zhao P., Zhao L., Bai L., Su Q., Feng Y., Ma W., Zhu J., Yang J., Zhang S. (2025). Colchicine Alleviates Interstitial Lung Disease in an Experimental Autoimmune Myositis Murine Model by Inhibiting the Formation of Neutrophil Extracellular Traps. Inflammation.

[44] Dupuis J., Sirois M.G., Rheaume E., Nguyen Q.T., Clavet-Lanthier M.E., Brand G., Mihalache-Avram T., Theberge-Julien G., Charpentier D., Rhainds D. (2020). Colchicine reduces lung injury in experimental acute respiratory distress syndrome. PLoS ONE.

[45] Spray L., Richardson G., Haendeler J., Altschmied J., Rumampouw V., Wallis S.B., Georgiopoulos G., White S., Unsworth A., Stellos K. (2025). Cardiovascular inflammaging: Mechanisms, consequences, and therapeutic perspectives. Cell Rep. Med..

[46] Ferrucci L., Fabbri E. (2018). Inflammageing: Chronic inflammation in ageing, cardiovascular disease, and frailty. Nat. Rev. Cardiol..

[47] Faust H.J., Zhang H., Han J., Wolf M.T., Jeon O.H., Sadtler K., Pena A.N., Chung L., Maestas D.R., Tam A.J. (2020). IL-17 and immunologically induced senescence regulate response to injury in osteoarthritis. J. Clin. Investig..

[48] Lopes-Paciencia S., Saint-Germain E., Rowell M.C., Ruiz A.F., Kalegari P., Ferbeyre G. (2019). The senescence-associated secretory phenotype and its regulation. Cytokine.

[49] Merino K.M., Jazwinski S.M., Rout N. (2021). Th17-type immunity and inflammation of aging. Aging.

[50] Bansal S.S., Ismahil M.A., Goel M., Zhou G., Rokosh G., Hamid T., Prabhu S.D. (2019). Dysfunctional and Proinflammatory Regulatory T-Lymphocytes Are Essential for Adverse Cardiac Remodeling in Ischemic Cardiomyopathy. Circulation.

[51] Tanaka K., Yoshioka K., Tatsumi K., Kimura S., Kasuya Y. (2014). Endothelin regulates function of IL-17-producing T cell subset. Life Sci..

[52] Li Q., Ding S., Wang Y.M., Xu X., Shen Z., Fu R., Liu M., Hu C., Zhang C., Cao Q. (2017). Age-associated alteration in Th17 cell response is related to endothelial cell senescence and atherosclerotic cerebral infarction. Am. J. Transl. Res..

[53] Andreotti F., Maggioni A.P., Campeggi A., Iervolino A., Scambia G., Massetti M. (2021). Anti-inflammatory therapy in ischaemic heart disease: From canakinumab to colchicine. Eur. Heart J. Suppl..

[54] Buckley C.D., Gilroy D.W., Serhan C.N. (2014). Proresolving lipid mediators and mechanisms in the resolution of acute inflammation. Immunity.

[55] Fouda R.T., Cherukury H.M., Kiven S.B., Garcia N.R., Argueta D.A., Velasco G.J., Gupta K., Roberts J.D. (2024). Colchicine reduces inflammation in a humanized transgenic murine model of sickle cell disease. Haematologica.

[56] Peng Y., Li Z., Zhang J., Dong Y., Zhang C., Dong Y., Zhai Y., Zheng H., Liu M., Zhao J. (2024). Low-Dose Colchicine Ameliorates Doxorubicin Cardiotoxicity Via Promoting Autolysosome Degradation. J. Am. Heart Assoc..

[57] Pierog J., Kubisa B., Grodzki T., Wojcik J., Pankowski J., Ostrowska J., Juzyszyn Z., Drozdzik M. (2007). Colchicine against ischemia-reperfusion injury in experimental lung transplantation. Ann. Transplant..

