# Supplementary material for: Colchicine Protects Against Sickle Cell Related Cardiomyopathy: Evidence of the Novel Role of Inflammaging
Source: Antioxidants (Basel). 2026 Jul 23;15(8):912. doi: 10.3390/antiox15080912 (PMC13509756; doi:10.3390/antiox15080912)

Supplementary Materials

1. Supplementary Methods
2. Supplementary Figures

1. Supplementary Methods

1.1 Echocardiography

Transthoracic echocardiography was performed with a Vevo 2100 echocardiograph (Visual Sonics, Toronto, Canada) equipped with a 22-55 MHz transducer (MicroScan Transducers, MS500D) as previously described [1]. Echocardiographic parameters were measured at the level of the papillary muscles in the parasternal short-axis view (M mode). Fractional shortening (FS) was calculated as previously described [2]. Diastolic parameters were measured with tissue Doppler and pulsed wave Doppler techniques in the apical long-axis view as reported in Schnelle M et al. [3]. Cardiac function was assessed when heart rate was 400-450 bpm.

1.2 Heart histological analysis

Paraffin-embedded tissue blocks were stained with: hematoxylin and eosin (H&E) for assessment of cardiomyocyte area, PicroSirius red for fibrosis evaluation and Perls' staining for iron content detection. Cardiomyocyte area and fibrosis were quantified as described previously [2]. Tissues were fixed in 10% neutral buffered formalin and embedded in paraffin. After the embedding, 3 μm-thick sections were cut and stained with hematoxylin and eosin (Bio-Optica, Italy) for histological examination. For immunohistochemical analysis, tissue sections were deparaffinized and rehydrated. For antigen retrieval, sections were subjected to heat-induced epitope retrieval (HIER) in 10 mM citrate buffer (pH 6.0). Slides were heated in a microwave oven for 20 minutes at maximum power and then allowed to cool for 10 minutes at room temperature. After cooling, sections were rinsed in cold distilled water prior to immunostaining. Endogenous peroxidase activity was quenched using 0.3% H_2O_2, and non-specific binding was blocked with 5% BSA for 30 minutes at room temperature. Sections were incubated overnight at +4°C with the following primary antibodies: anti-IL-17A rabbit polyclonal antibody (Thermofisher, PA5-114455) and anti-MPO rabbit polyclonal antibody (Thermofisher, PA5-16672) in PBS/Triton 0.1%. After washing in PBS, sections were incubated with a species-appropriate HRP-conjugated anti-rabbit secondary antibody (Thermo Fisher Scientific) for 30 minutes at room temperature, protected from light. Signal detection was performed using DAB. Nuclei were counterstained with hematoxylin, and sections were mounted using a resin-based mounting medium. Negative controls sections were processed in parallel without the primary antibody. Images were acquired using a Leica DM6 microscope (Leica microsystems) equipped with a digital camera, using a 20× objective for quantitative analysis and a 10× objective for representative images. Representative images were selected based on staining quality and anatomical region. Quantitative analysis of IL-17A- and MPO-positive cells was performed using ImageJ/Fiji software. Three non-overlapping fields per section were acquired, and twenty-five randomly selected regions per field were quantified in a blinded manner. Measurements were averaged per field and then across fields to obtain a single value per heart slice.

1.3 Flow cytometric analysis of Th17 cells. Th17 heart, spleen infiltrate.

PBS perfused hearts were disaggregated using the Gentle MACs disaggregator (Miltenyi Biotec) and TH17 cells were evaluated using the Mouse Th1/Th2/Th17 Phenotyping Kit (BD Biosciences, New Jersey, USA), following the manufacturer instructions (Technical Data Sheet, Mouse Th1/Th2/Th17 Phenotyping Kit, BD Biosciences) [4]. Before acquisition 5 μl of counting beads (CountBright^T Absolute Counting Beads, ThermoFisher Scientific) were added to each sample. Samples were acquired using BD LSRFortessa (BD Biosciences) Analysis of acquired samples were performed using *FlowJo* Software (Tree Star, Ashland, OR; USA) [1].

1.3.1 Peripheral Th17 cells. Circulating Th17 cells were identified in freshly collected blood by flow cytometry analysis using Mouse Th1/Th2/Th17 Phenotyping Kit (BD Biosciences, New Jersey, USA), following the manufacturer instructions.

1.4 *Heart immunoblot analysis, immunoprecipitation assay.*

Frozen hearts were homogenized and lysed with iced lyses buffer (150 mM NaCl, 25 mM bicine, 0.1% SDS, 2% Triton X-100, 1 mM EDTA, protease inhibitor cocktail tablets (Roche), 1 mM Na3VO4 final concentration) then centrifuged 30 min at 4°C at 12,000 g.[5,6] Specific antibodies used are: anti-PDGF Receptor, anti-FGF Receptor (phospho Tyr653/654), anti-PDGF Receptor (phospho Tyr740) from GeneTex (Ca, USA); Anti-GAPDH, Anti-SERCA-2 (F-1), anti-TGF-β Receptor, , anti-ET-1, , anti-IL-6; anti-heme oxygenase 1(HO-1) from Santa Cruz Biotechnology (CA, USA); anti-Phospho (Ser536) NF-kB p65, anti-NF-kB p65 (clone C22B4); anti-ATG5 from Cell Signaling Technology (Danvers, MA, USA); anti-P-Selectin; Anti- PDGF-BB; anti-Caspase 1; anti pro-Caspase 3 (clone E83-103); anti Nrf2-phospho-S40 (Clone EP1809Y); anti Nrf2; anti-SQTM1 / P62; anti-LC3A/B from Abcam (Cambridge, UK); anti-FGF Receptor from Thermo Fisher Scientific (Waltham, MA, USA) ;; anti-VCAM-1 from R and D Systems (Minneapolis, MN, USA); anti-NLRP3 form Adipogen AG (Fuellinsdorf, Switzerland); Anti-Thromboxane synthase TBXS-1 was from Cayman Chemical Company (Michigan, USA); Anti-Phospho-Smad 2; anti-Smad2; anti-Phospho-Smad 3; anti-Smad 3; anti-Smad 4; anti-Smad 7 form ABclonal Inc (Woburn, MA, USA); anti-Atg7 (APG7, ProSci Inc's, USA). Secondary donkey anti-rabbit IgG (dilution 1:10000) and anti-mouse IgG (dilution 1:5000) HRP conjugated were from GE Healthcare Life Sciences (Little Chalfont, UK); secondary donkey anti goat IgG (dilution 1:10000) HRP conjugated was from SCBT, secondary donkey anti rat IgG (dilution 1:5000) HRP conjugated was from AbCam. Blots were developed with Luminata Forte Chemiluminescent HRP Substrate from Merk Millipore (Burlington, MA, USA), and images were acquired with the Alliance Q9 Advanced imaging system (Uvitec, UK). Densitometric analyses were performed with the Nine Alliance software (Uvitec, UK). Immunoprecipitations (IP) assays of anti-phospho-Tyr proteins from heart lysate were carried out using the Protein A Agarose (Thermo Fisher scientific, USA) and the mix of the anti phospho-Tyrosine monoclonal antibodies PY99 (Santa Cruz Biotechnology, USA) and 4G10 (Merck Group, De) [1].

1.5 *Heart molecular analysis.*

For nucleic acid extraction, hearts snap frozen in liquid nitrogen following excision were homogenized in RNA lysis buffer (Zymo Research) using the GentleMACStissue dissociator (Miltenyi Biotec), and clear lysates were loaded onto silica spin columns for RNA/miRNA purification following the manufacturer's protocol. The extracted nucleic acids were analysed as previously described by Federti et al. (1).

2. Supplementary references

1. Federti, E.; Mattoscio, D.; Recchiuti, A.; Matte, A.; Monti, M.; Cozzolino, F.; Iezzi, M.; Ceci, M.; Ghigo, A.; Tolosano, E.; et al. 17(R)-Resolvin D1 Protects against Sickle Cell-Related Inflammatory Cardiomyopathy in Humanized Mice. *Blood* **2025**, doi:10.1182/blood.2024024768.
2. Li, M.; Sala, V.; De Santis, M.C.; Cimino, J.; Cappello, P.; Pianca, N.; Di Bona, A.; Margaria, J.P.; Martini, M.; Lazzarini, E.; et al. Phosphoinositide 3-Kinase Gamma Inhibition Protects From Anthracycline Cardiotoxicity and Reduces Tumor Growth. *Circulation* **2018**, *138*, 696-711, doi:10.1161/CIRCULATIONAHA.117.030352.
3. Schnelle, M.; Catibog, N.; Zhang, M.; Nabeebaccus, A.A.; Anderson, G.; Richards, D.A.; Sawyer, G.; Zhang, X.; Toischer, K.; Hasenfuss, G.; et al. Echocardiographic evaluation of diastolic function in mouse models of heart disease. *J Mol Cell Cardiol* **2018**, *114*, 20-28, doi:10.1016/j.yjmcc.2017.10.006.
4. Aldo, P.; Marusov, G.; Svancara, D.; David, J.; Mor, G. Simple Plex() : A Novel Multi-Analyte, Automated Microfluidic Immunoassay Platform for the Detection of Human and Mouse Cytokines and Chemokines. *Am J Reprod Immunol* **2016**, *75*, 678-693, doi:10.1111/aji.12512.
5. Matte, A.; Recchiuti, A.; Federti, E.; Koehl, B.; Mintz, T.; El Nemer, W.; Tharaux, P.L.; Brousse, V.; Andolfo, I.; Lamolinara, A.; et al. Resolution of sickle cell disease-associated inflammation and tissue damage with 17R-resolvin D1. *Blood* **2019**, *133*, 252-265, doi:10.1182/blood-2018-07-865378.

6. Federti, E.; Vinchi, F.; Iatcenko, I.; Ghigo, A.; Matte, A.; Toya, S.C.M.; Siciliano, A.; Chiabrando, D.; Tolosano, E.; Vance, S.Z.; et al. Duality of Nrf2 in iron-overload cardiomyopathy. *Haematologica* **2023**, *108*, 1335-1348, doi:10.3324/haematol.2022.281995.

3. Supplementary Table

Table S1. Echocardiographic parameters of sickle cell (SS) mice treated with either vehicle or colchicine (CLC)

	Vehicle treated SS mice	CLC treated SS mice
n	4	9
FS (%)	41.1±2.9	39.1±1.3
EF (%)	71.6±3.5	69.5±1.7
IVSD (mm)	0.86±0.06	0.90±0.04
IVSS (mm)	1.31±0.08	1.27±0.06
LVPWD (mm)	1.19±0.09	1.04±0.03
LVPWS (mm)	1.64± 0.02	1.46±0.05
LVIDD (mm)	4.62± 0.24	4.55±0.16
LVIDS (mm)	2.71± 0.11	2.78±0.14
LVW (mg)	171.3± 27.4	153.2±11.1
LVEDV (µl)	99.7±12.0	103.7± 7.8
LVESV (µl)	19.5±2.7	24.8±1.9
LVOT CO (ml/min)	57.7±16.0	35.0±8.0
LVOT SV (ml)	156.8±43.1	92.2±11.1
RVOT CO (ml/min)	29.3±2.7	16.5±2.3**
RVOT SV (ml)	79.3±6.7	62.4±7.1
MPI	0.51±0.09	0.61±0.07
IVRT (ms)	15.5±3.3	32.6±3.6**
MVDT (ms)	16.7±1.4	27.1±2.1**
MV E/A	1.56±0.13	1.78±0.15
MVDT (ms)	4	9
MV E/A	41.1±2.9	39.1±1.3
n	71.6±3.5	69.5±1.7
FS (%)	0.86±0.06	0.90±0.04
EF (%)	1.31±0.08	1.27±0.06
IVSD (mm)	1.19±0.09	1.04±0.03

Data are presented as mean ± SEM. ***P*<0.01 by unpaired Student's t test. Abbreviations: FS, fractional shortening; EF, ejection fraction; IVSD, interventricular septal thickness at end-diastole; IVSS, interventricular septal thickness at end-systole; LVPWD, left ventricular posterior wall thickness at end-diastole; LVPWS, left ventricular posterior wall thickness at end-systole; LVEDD, left ventricular end-diastolic diameter; LVEDS, left ventricular end-systolic diameter; LVM, left ventricle mass; LVEDV, left ventricle end-diastolic volume; LVESV, left ventricle end-systolic volume; LVOT CO, left ventricle outflow tract cardiac output; LVOT SV, left ventricle outflow tract stroke volume; RVOT CO, right ventricle outflow tract cardiac output; RVOT SV, left ventricle outflow tract stroke volume; IVRT, isovolumetric relaxation time; MPI, myocardial performance index; MVDT, mitral valve deceleration time; MV E/A, mitral valve E/A.

4. Supplementary Figures

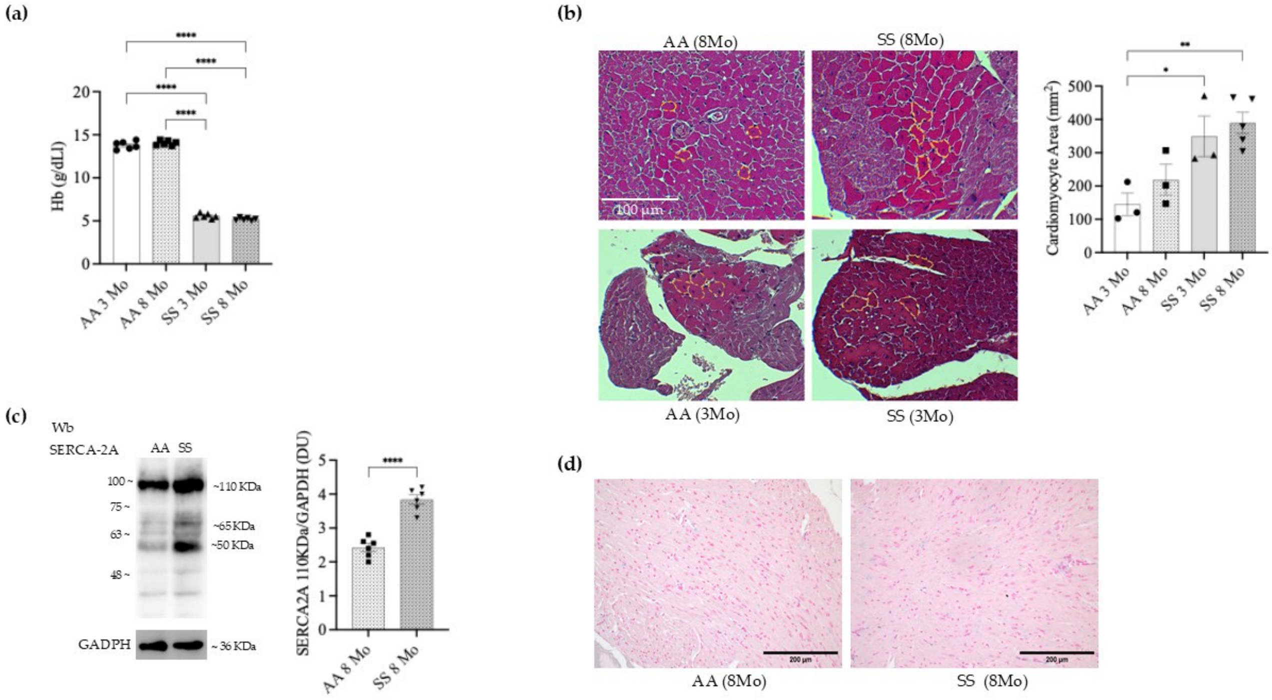

Figure S1. Hemoglobin, cardiomyocyte area and SERCA2A expression aging sickle cell mice.
a. Hemoglobin (Hb) in healthy (AA) and sickle cell (SS) mice at 3 and 8 months of age. **b.** Representative images of hematoxylin & eosin (H7E) staining and relative quantification of cardiomyocyte area in heart sections from AA and SS mice at 3 and 8 months of age. Representative cardiomyocyte areas are shown by dashed lines. **c.** Immunoblot analysis using specific antibodies against SERCA2A in heart from of healthy (AA) and sickle (SS) mice. 75 μg/μL of protein loaded on an 8% T, 2.5%C polyacrylamide gel. GAPDH serves as protein loading control. One representative gel from 6 with similar results is shown. Densitometric analysis of immunoblots is shown on the right. **d.** Representative heart sections from AA and SS mice stained with Perls' reaction. Data in the column charts are presented as individual data points with mean ± SEM. Significance codes: *p < 0.05; **p < 0.01; ***p < 0.001; ****p < 0.0001; not significant comparisons were not reported. Adjusted p-values were used when multiple comparisons were involved.

(a)

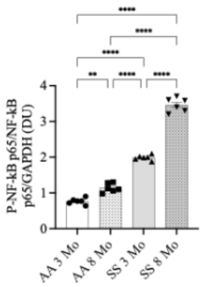

(b)

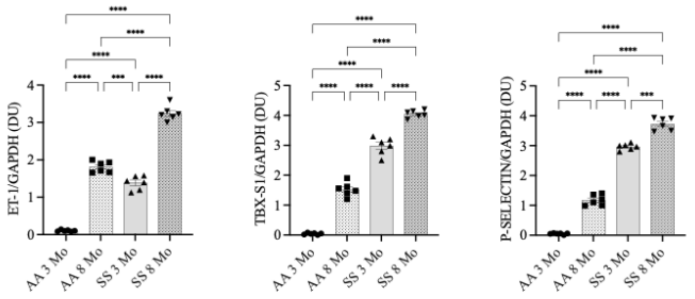

Figure S2. Densitometric analysis of Figure 2a. a. Densitometric analysis of immunoblots of Figure 2a. **b.** Densitometric analysis of immunoblots from Figure 2b Data in the column charts are presented as individual data points with mean ± SEM. Significance codes: *p < 0.05; **p < 0.01; ***p < 0.001; ****p < 0.0001; not significant comparisons were not reported. Adjusted p-values were used when multiple comparisons were involved.

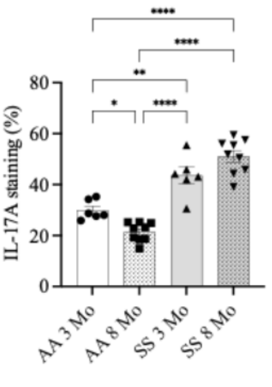

Figure S3. Quantification myeloperoxidase and gating strategy for Th17 lymphocytes. Quantification of IL-17A staining related to Figure 3c. Data in the column charts are presented as individual data points with mean ± SEM. Significance codes: *p < 0.05; **p < 0.01; ***p < 0.001; ****p < 0.0001; not significant comparisons were not reported. Adjusted p-values were used when multiple comparisons were involved.

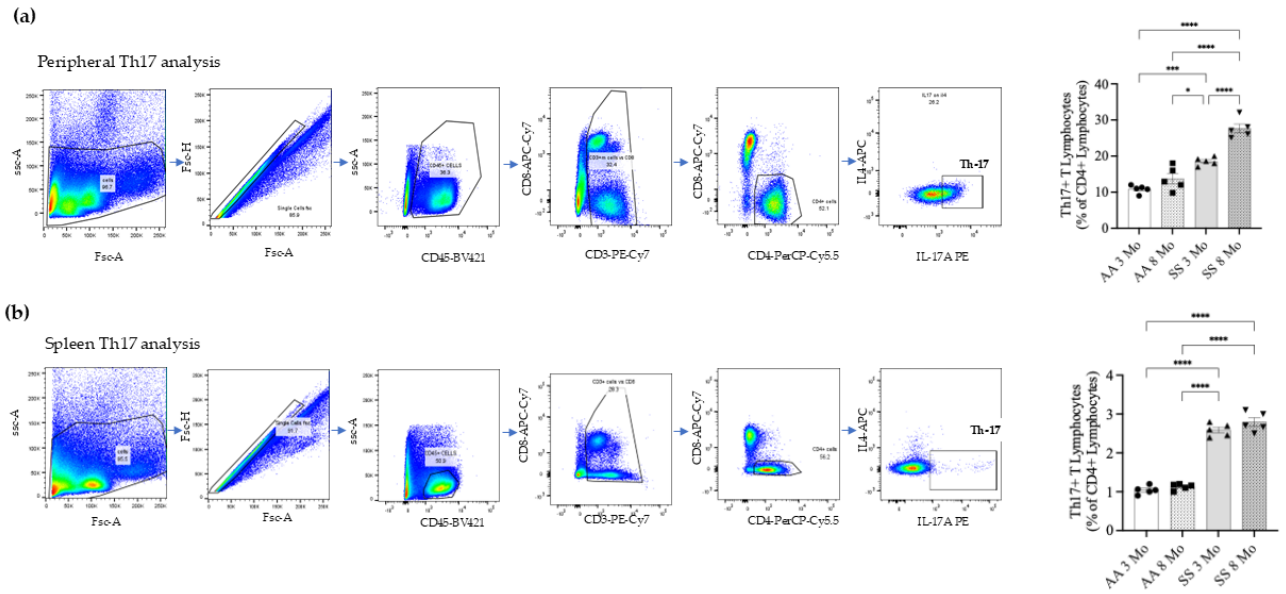

Figure S4. Gating strategy and quantification of peripheral and splenic Th17 lymphocytes. Upper panel. Gating strategy for peripheral Th17 cells **(left)** and Th17 **(right)** in healthy (AA) and sickle (SS) mice at 3 and 8 months of age. **Lower panel.** Gating strategy for splenic Th17 cells **(left)** and Th17 **(right)** in healthy (AA) and sickle (SS) mice at 3 and 8 months of age. Data in the column charts are presented as individual data points with mean ± SEM. Significance codes: *p < 0.05; **p < 0.01; ***p < 0.001; ****p < 0.0001; not significant comparisons were not reported. Adjusted p-values were used when multiple comparisons were involved.

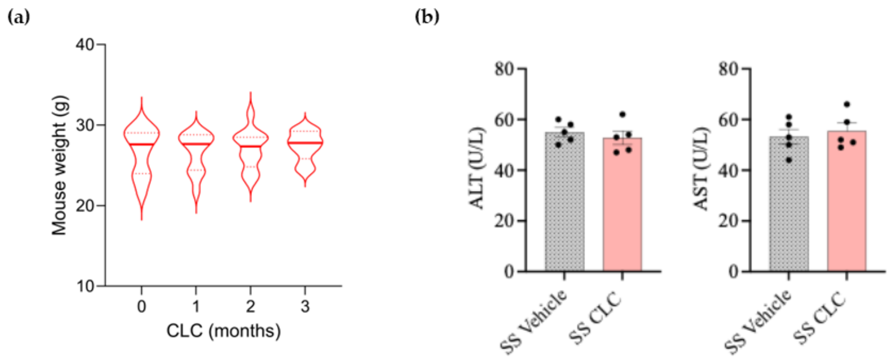

Figure S5. Mouse weight and liver enzymes in sickle cell mice treated with either vehicle or colchicine. a. Weight of sickle cell (SS) mice during colchicine (CLC) treatment (n=12). **b.** Liver enzymes (alanine aminotransferase, ALT and aspartate aminotransferase, AST) in SS mice treated with either vehicle or colchicine (CLC). Data in the column charts are presented as individual data points with mean ± SEM. Significance codes: *p < 0.05; **p < 0.01; ***p < 0.001; ****p < 0.0001; not significant comparisons were not reported.

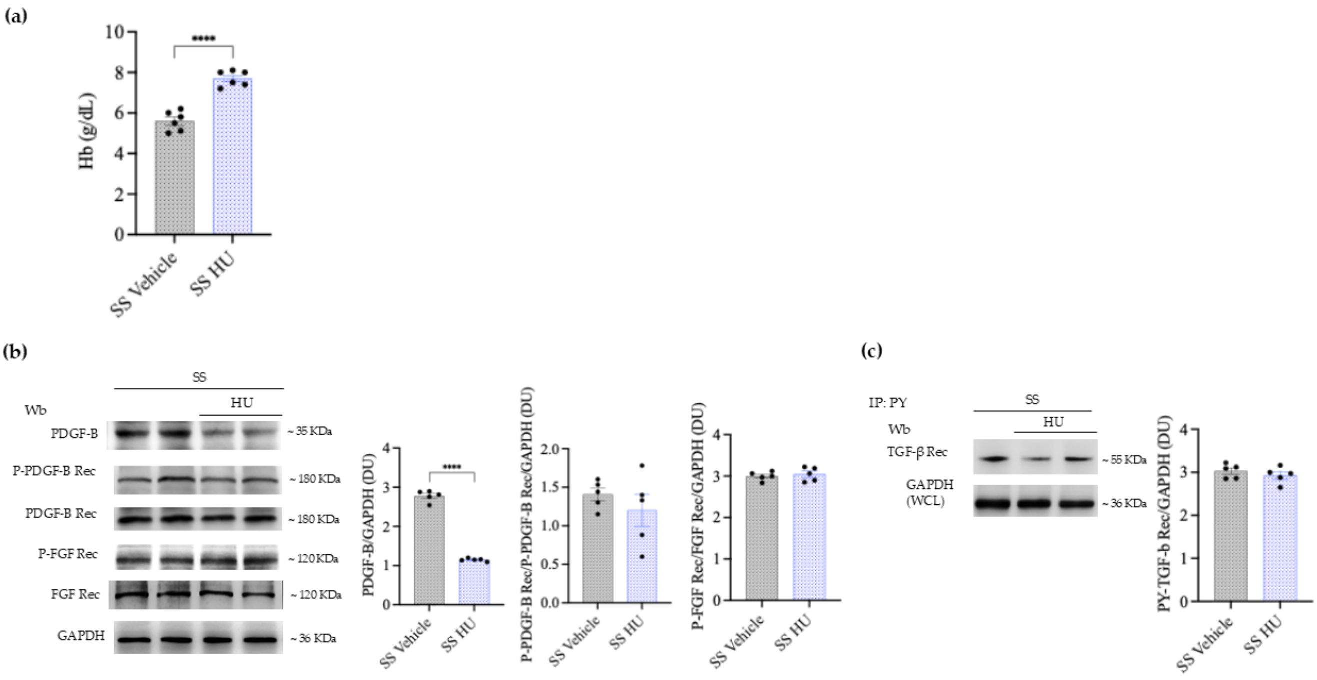

Figure S6. Hemoglobin, heart expression and degree of activation of PDGF-B/FGF-receptors. a. Hemoglobin (Hb) in sickle cell (SS) mice treated with either vehicle or hydroxyurea (HU). **b.** Immunoblot analysis, using specific antibodies against PDGFB, phosphorylated p-PDGFR-B, PDGFR-B, P-FGFR, and FGFR in heart from SS mice treated as with either vehicle or HU. 75 µg/µL of protein loaded on an 8% T, 2.5%C polyacrylamide gel. GAPDH serves as protein loading control. One representative gel from 5 with similar results is shown. Densitometric analysis of immunoblots is shown on the bottom. **c.** Immunoprecipitation from heart of SS mice treated as in b, using specific anti-phospho-Tyrosine antibodies (IP: PY), revealed with specific anti-TGF β receptor (Rec) antibody (75 µg/µL of protein loaded on an 8% T, 2.5%C polyacrylamide gel). GAPDH in whole cell lysate (WCL) is used as loading controls. Densitometric analysis of immunoblots is shown on the right Data in the column charts are presented as individual data points with mean ± SEM. Significance codes: *p < 0.05; **p < 0.01; ***p < 0.001; ****p < 0.0001; not significant comparisons were not reported.

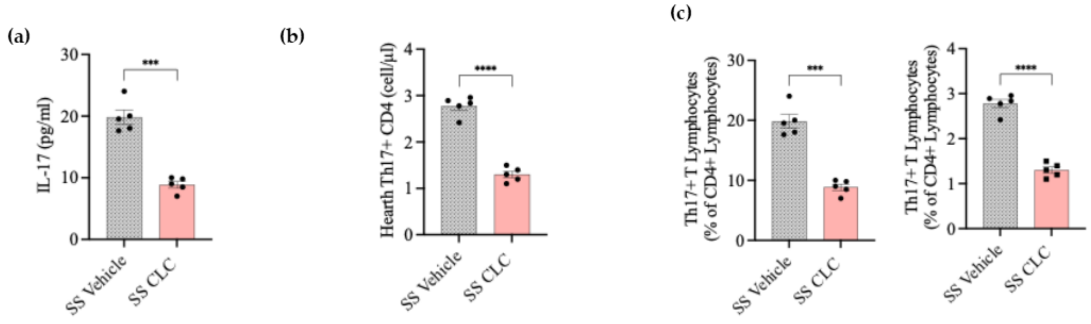

Figure S7. Plasma IL17A, heart Th17 lymphocytes infiltration, peripheral and splenic Th17 lymphocytes. a. plasma IL17A in sickle cell (SS) mice treated with either vehicle or colchicine (CLC). **b** Heart Th17 infiltrates in sickle cell (SS) mice treated with either vehicle or colchicine (CLC). **c.** peripheral (left panel) and splenic (right panel) Th17 cells in sickle cell (SS) mice treated with either vehicle or colchicine (CLC). Data in the column charts are presented as individual data points with mean ± SEM. Significance codes: *p < 0.05; **p < 0.01; ***p < 0.001; ****p < 0.0001; not significant comparisons were not reported.

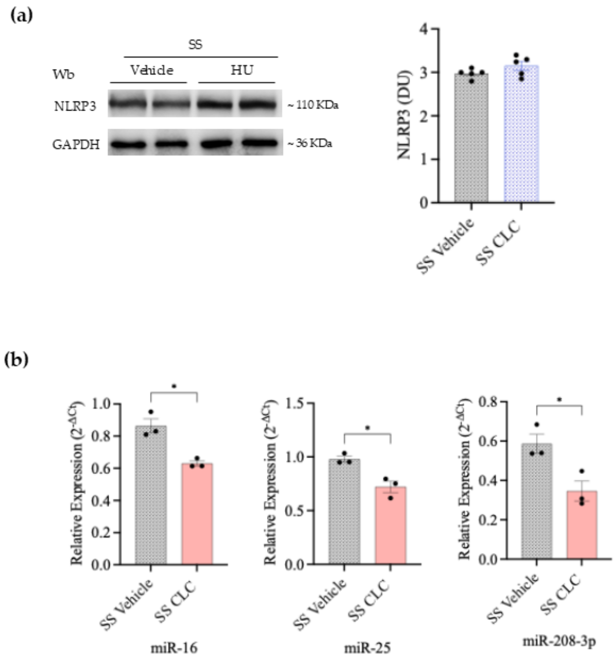

Figure S8. Heart expression of NLRP3 and pro-resolving miRNAs. a. Immunoblot analysis, using specific antibodies against NLRP3 in heart from SS mice treated with either vehicle or hydroxyurea (HU). 75 µg/µL of protein loaded on an 8% T, 2.5%C polyacrylamide gel. GAPDH serves as protein loading control. One representative gel from 6 with similar results is shown. Densitometric analysis of immunoblots is shown in the lower panel. **b.** Pro-resolving miRNAs regulated by colchicine in SS mice. miRNA expression was determined with qPCR as we published. Relative expression of each miRNA was calculated the ΔCt method using RNU6 as housekeeping (for controlling equal cDNA input). Each sample was assayed as a triplicate. Data in the column charts are presented as individual data points with mean ± SEM. Significance codes: *p < 0.05; **p < 0.01; ***p < 0.001; ****p < 0.0001; not significant comparisons were not reported.

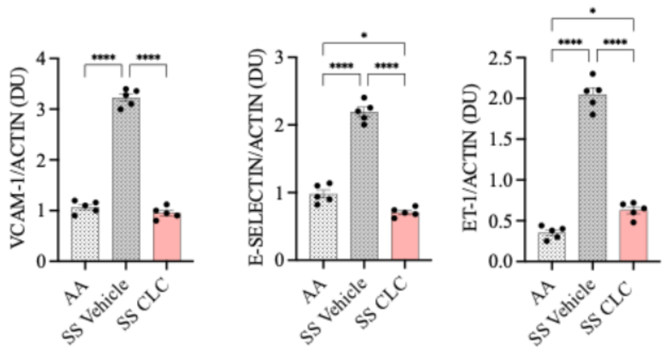

Figure S9. Densitometric analysis of immunoblots in Figure 7f. Data in the column charts are presented as individual data points with mean ± SEM. Significance codes: ns, not significant; *p < 0.05; **p < 0.01; ***p < 0.001; ****p < 0.0001.

The figures below provide the raw and uncropped images of all Western blot experiments presented in this study.

Fig 1 d

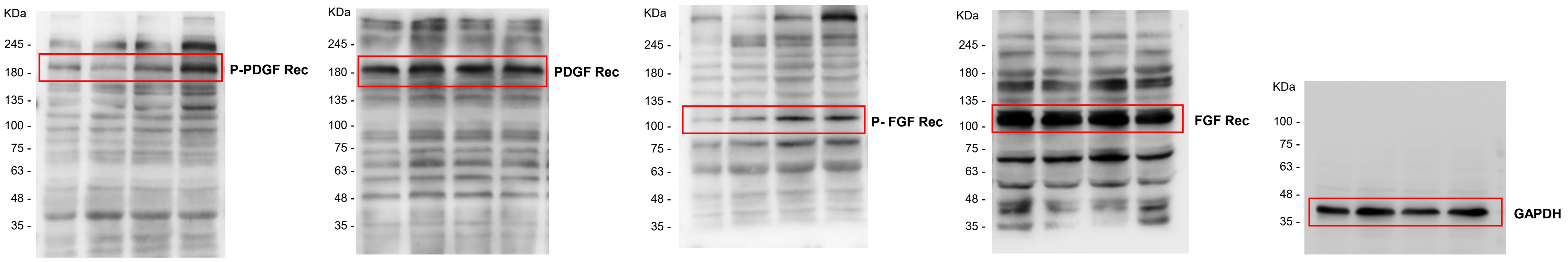

Fig 1 e

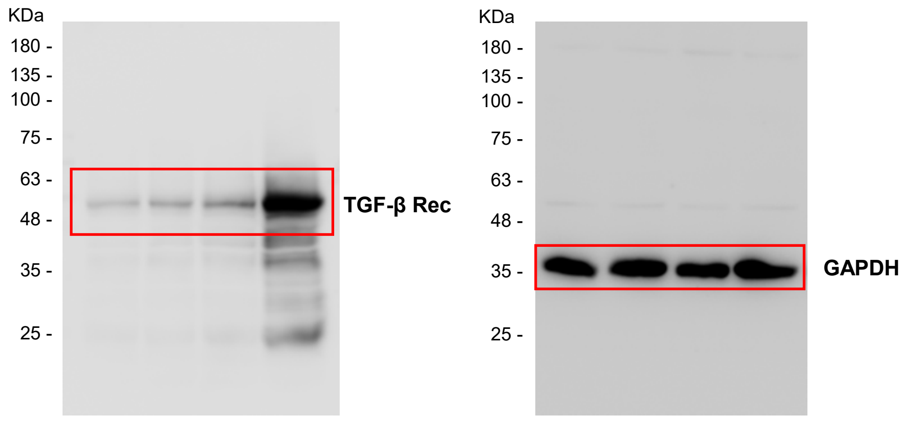

Fig 2 a

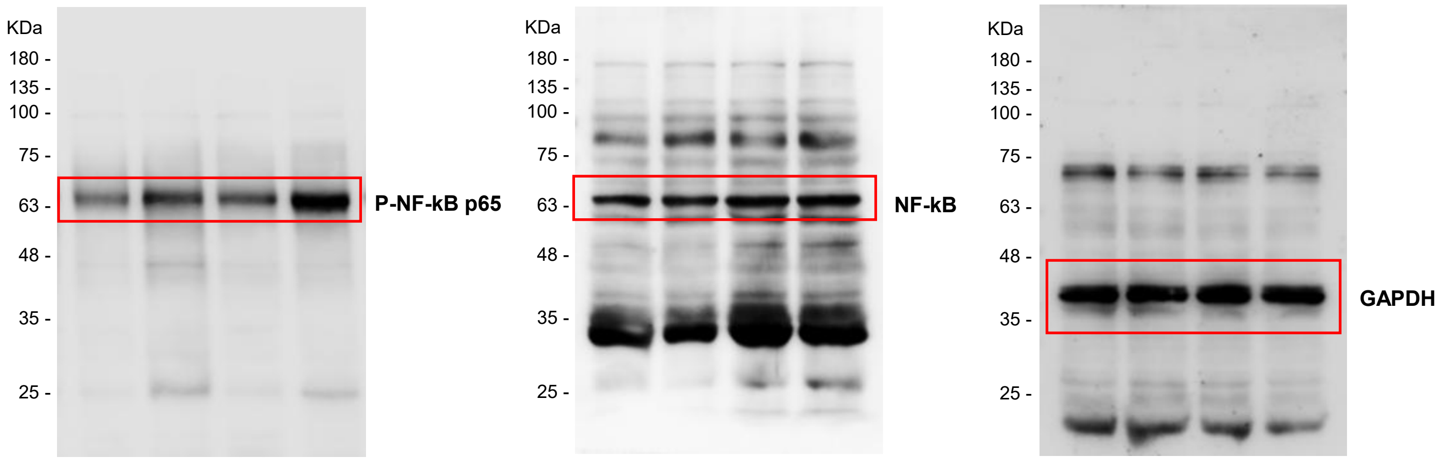

Fig 2 b

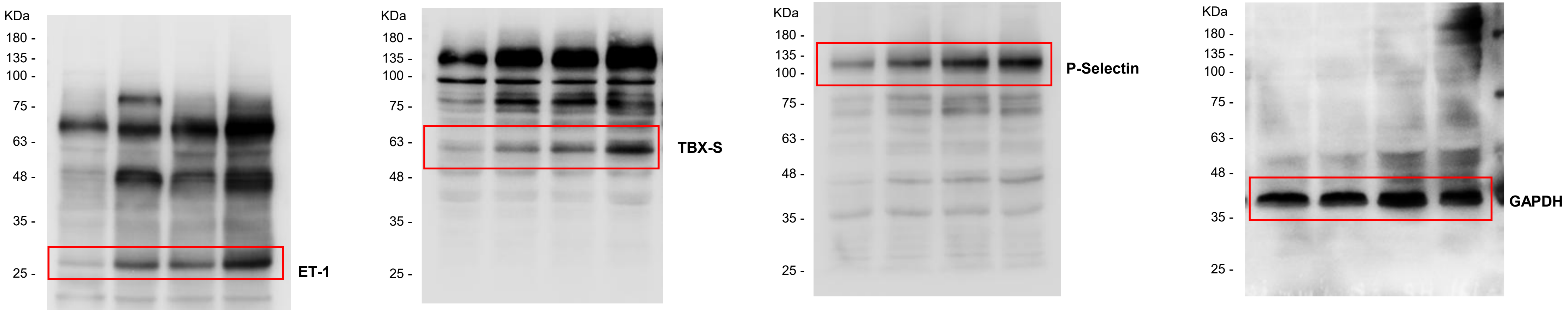
KDa
180 -
135 -
100 -
75 -
63 -
48 -
35 -
25 -
ET-1
TBX-S
P-Selectin
GAPDH

Fig 4 c

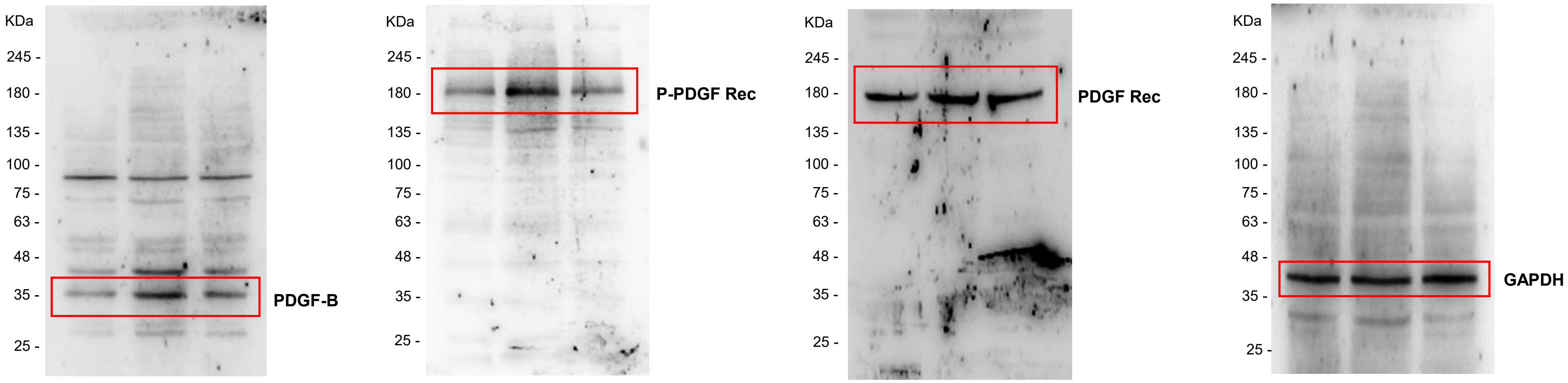
KDa
245 -
180 -
135 -
100 -
75 -
63 -
48 -
35 -
25 -
PDGF-B
P-PDGF Rec
PDGF Rec
GAPDH

Fig 4 d

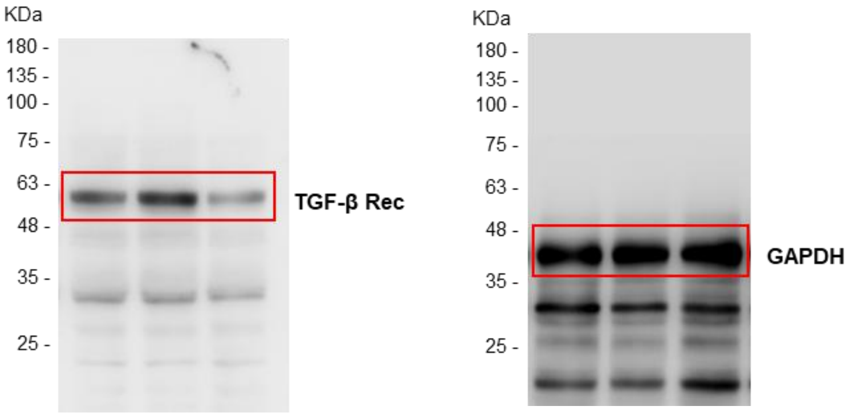

Fig 4 e

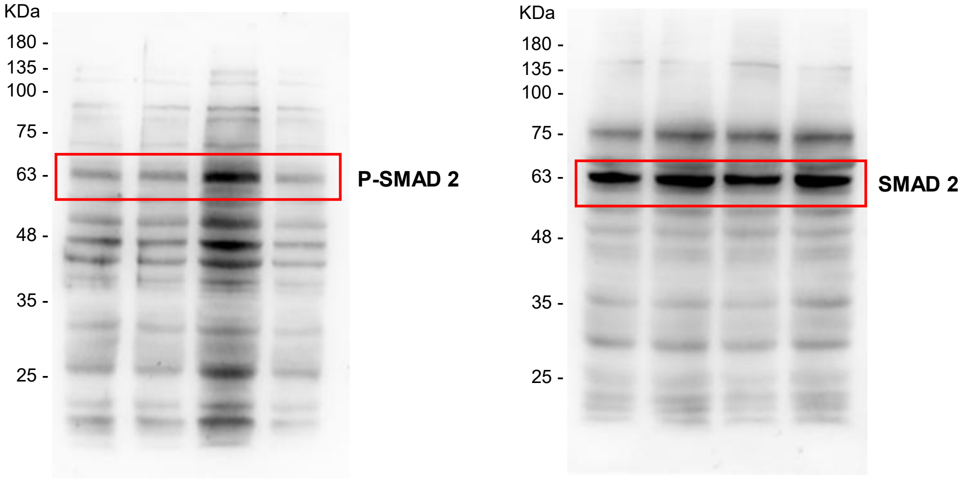

Fig 4 e

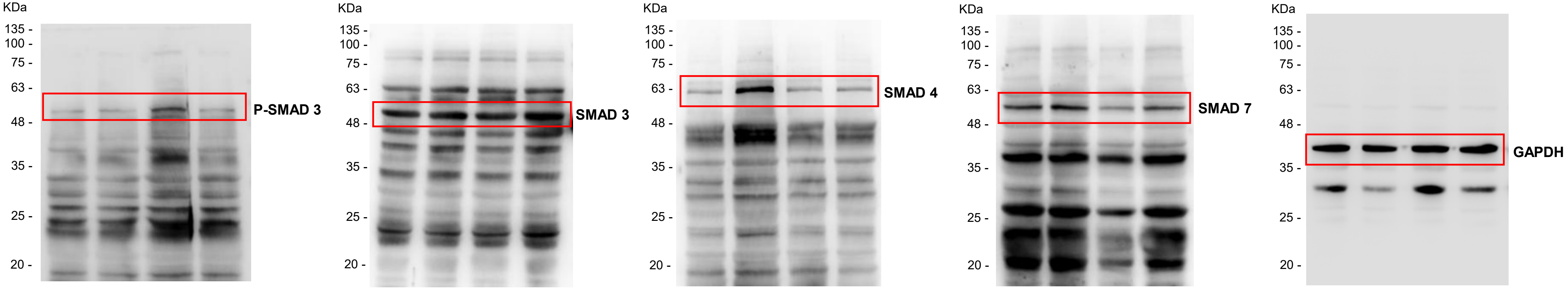

Fig 5 c

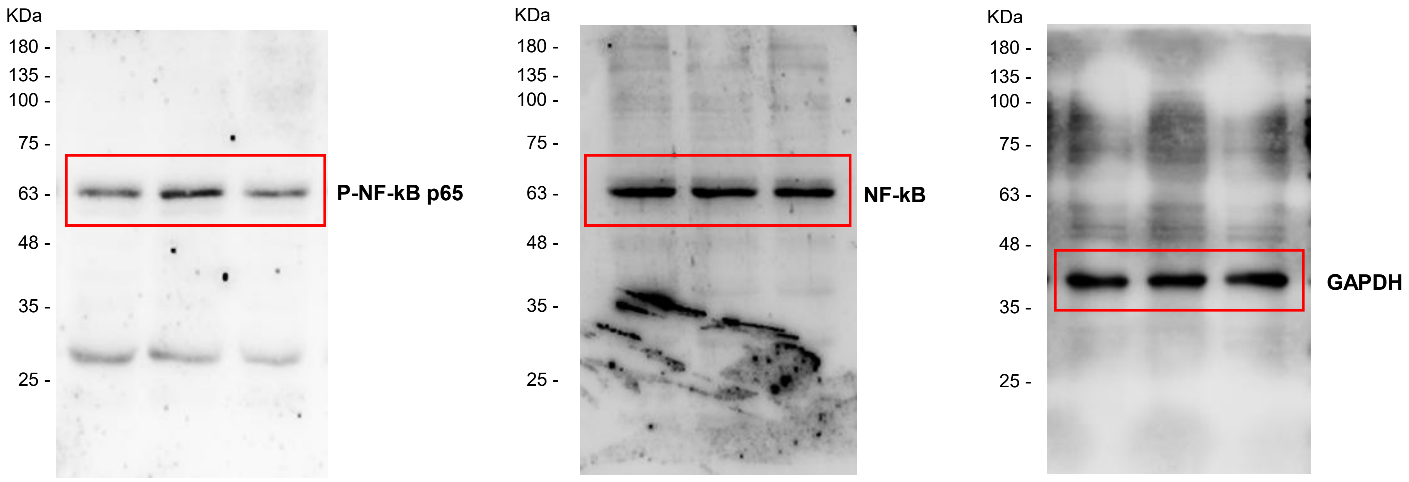
KDa
180 -
135 -
100 -
75 -
63 -
48 -
35 -
25 -
P-NF-kB p65
NF-kB
GAPDH

Fig 5 d

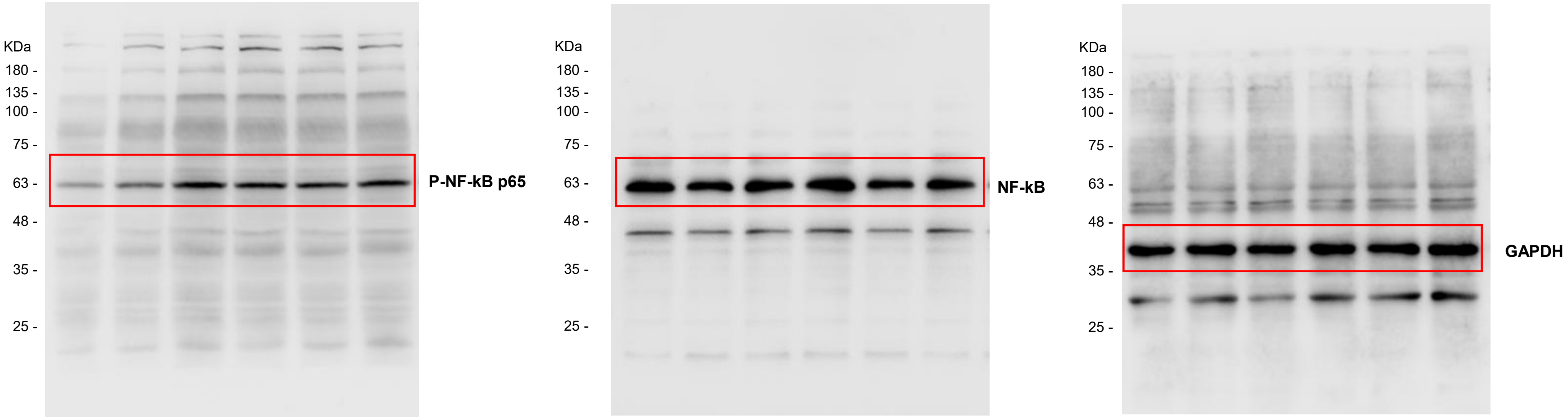
KDa
180 -
135 -
100 -
75 -
63 -
48 -
35 -
25 -
P-NF-kB p65
NF-kB
GAPDH

Fig 6 a

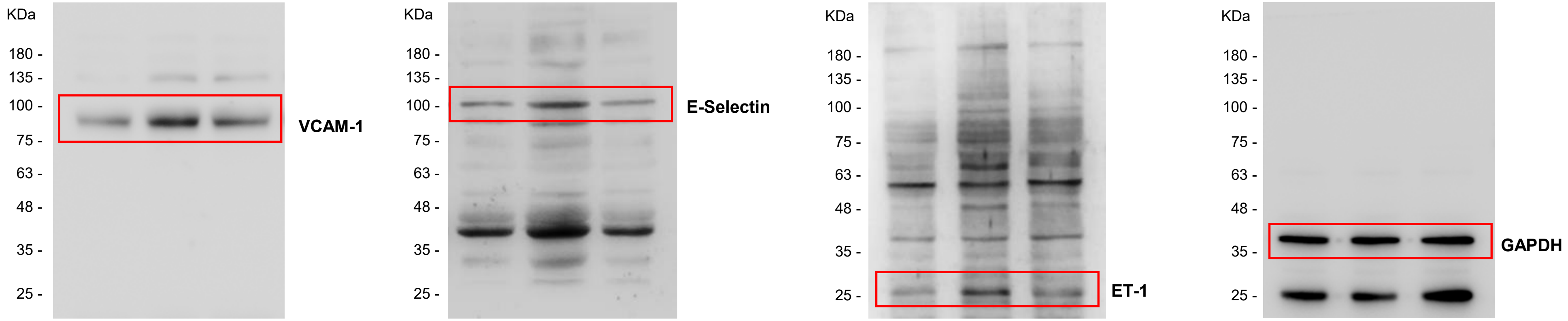
KDa
180 -
135 -
100 -
75 -
63 -
48 -
35 -
25 -
VCAM-1
E-Selectin
ET-1
GAPDH

Fig 6 b

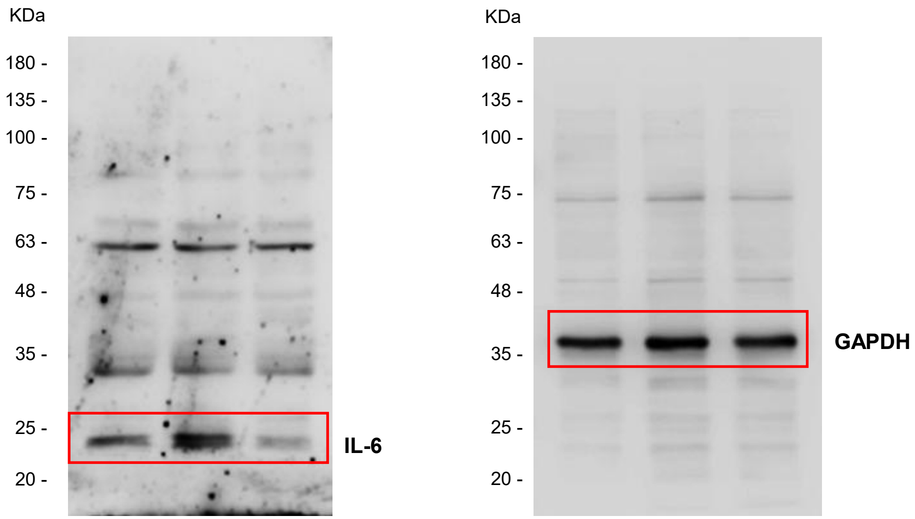
KDa
180 -
135 -
100 -
75 -
63 -
48 -
35 -
25 -
20 -
IL-6
GAPDH

Fig 6 c

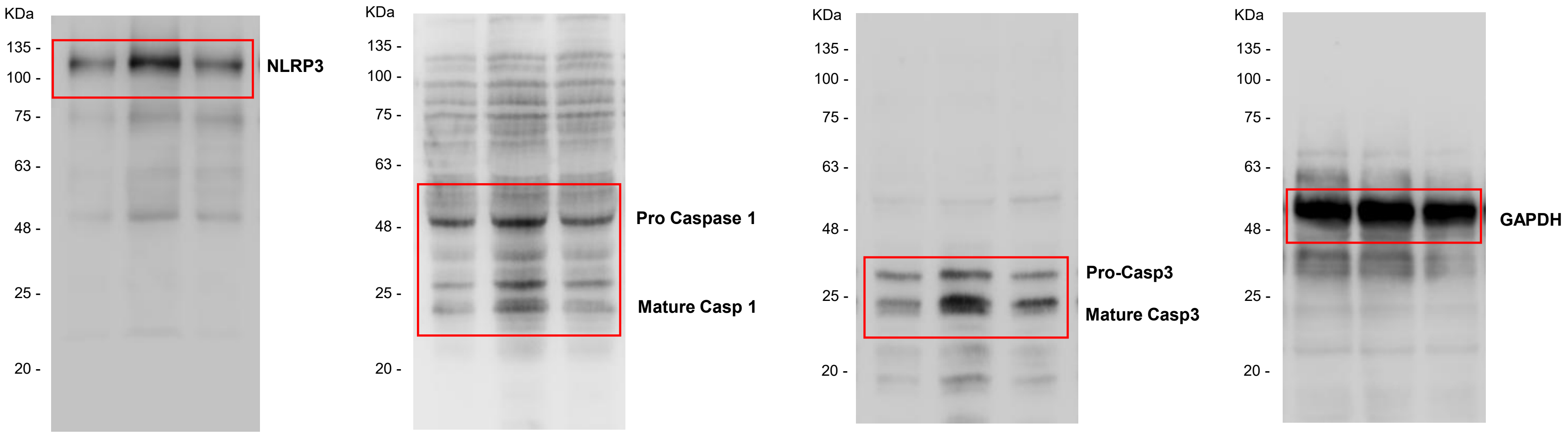
KDa
135 -
100 -
75 -
63 -
48 -
25 -
20 -
NLRP3
Pro Caspase 1
Mature Casp 1
Pro-Casp3
Mature Casp3
GAPDH

Fig 6 d

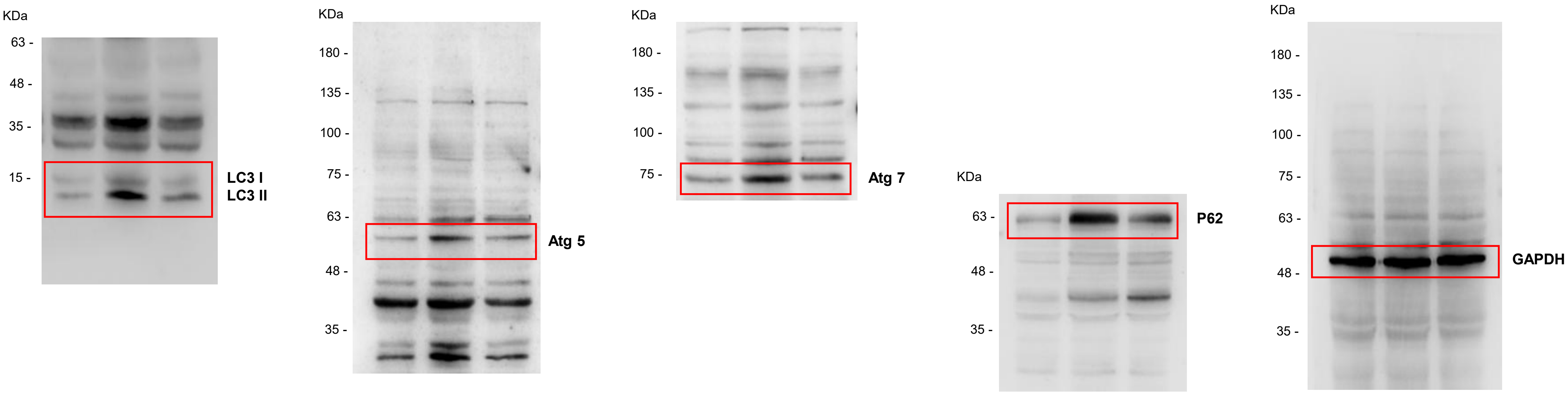
KDa
180 -
135 -
100 -
75 -
63 -
48 -
35 -
15 -
LC3 I
LC3 II
Atg 5
Atg 7
P62
GAPDH

Fig 6 b

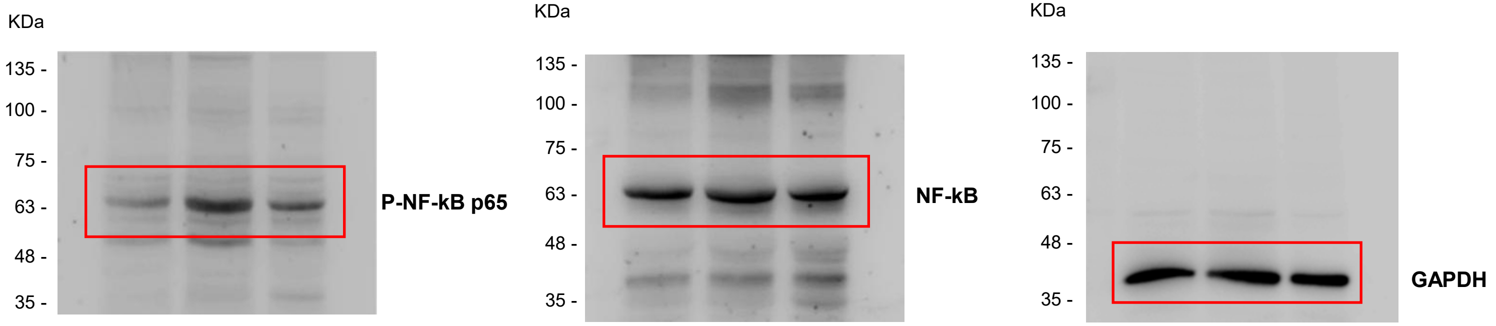
KDa
135 -
100 -
75 -
63 -
48 -
35 -
P-NF-kB p65
KDa
135 -
100 -
75 -
63 -
48 -
35 -
NF-kB
KDa
135 -
100 -
75 -
63 -
48 -
35 -
GAPDH

Fig 6 c

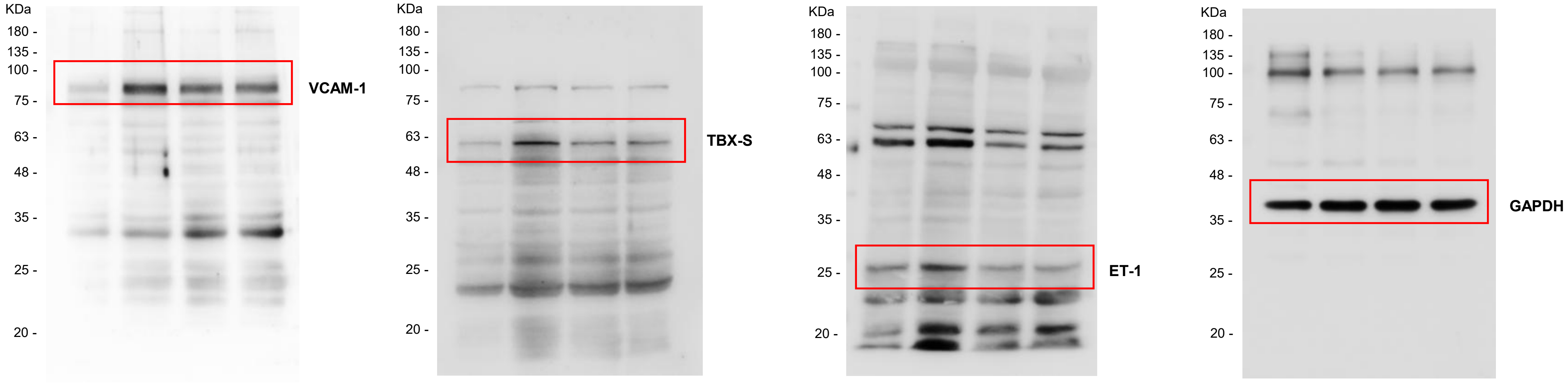
KDa
180 -
135 -
100 -
75 -
63 -
48 -
35 -
25 -
20 -
VCAM-1
KDa
180 -
135 -
100 -
75 -
63 -
48 -
35 -
25 -
20 -
TBX-S
KDa
180 -
135 -
100 -
75 -
63 -
48 -
35 -
25 -
20 -
ET-1
KDa
180 -
135 -
100 -
75 -
63 -
48 -
35 -
25 -
20 -
GAPDH

Fig 7 d

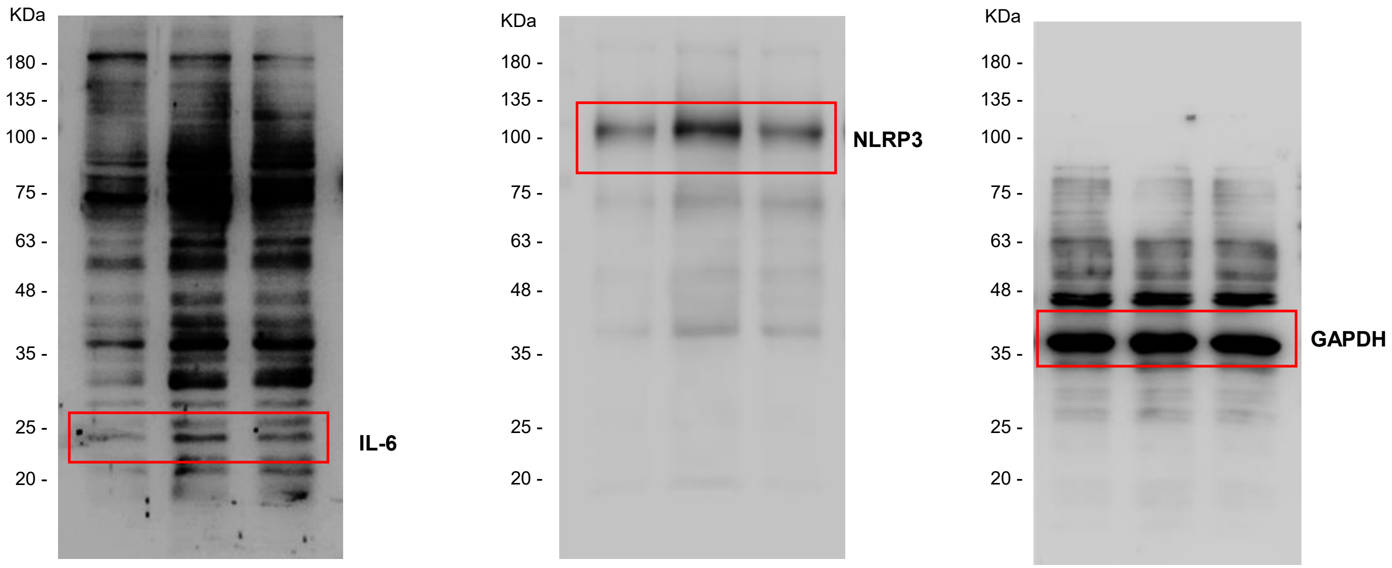
KDa
180 -
135 -
100 -
75 -
63 -
48 -
35 -
25 -
20 -
IL-6
NLRP3
GAPDH

Fig 7 e

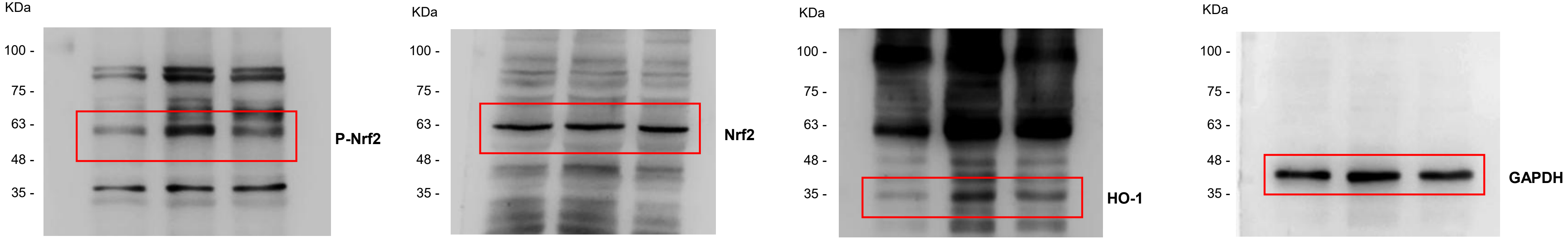
KDa
100 -
75 -
63 -
48 -
35 -
P-Nrf2
Nrf2
HO-1
GAPDH

Fig 7 F

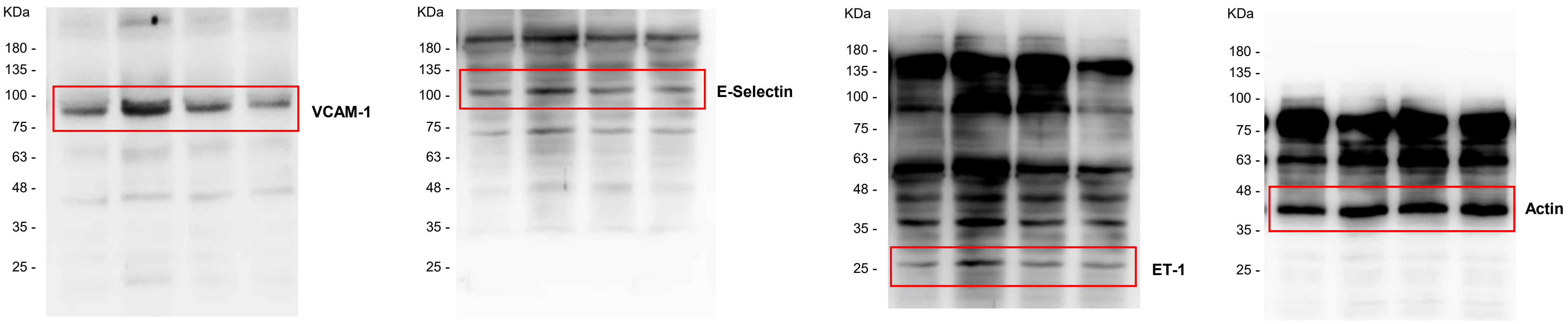

Fig S1 d

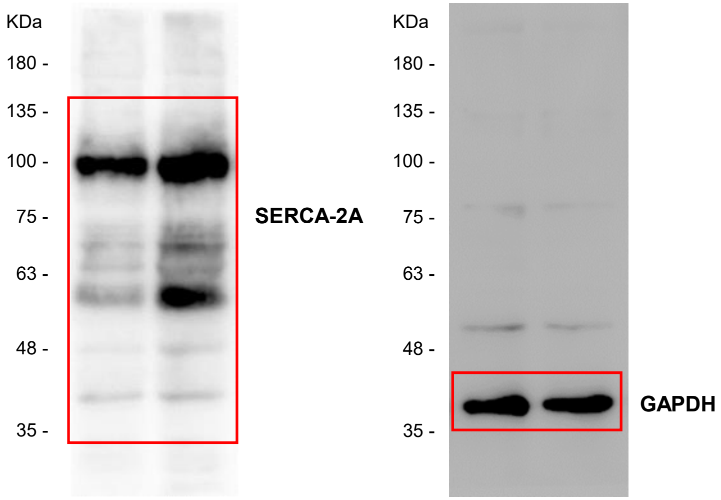

Fig S6 c

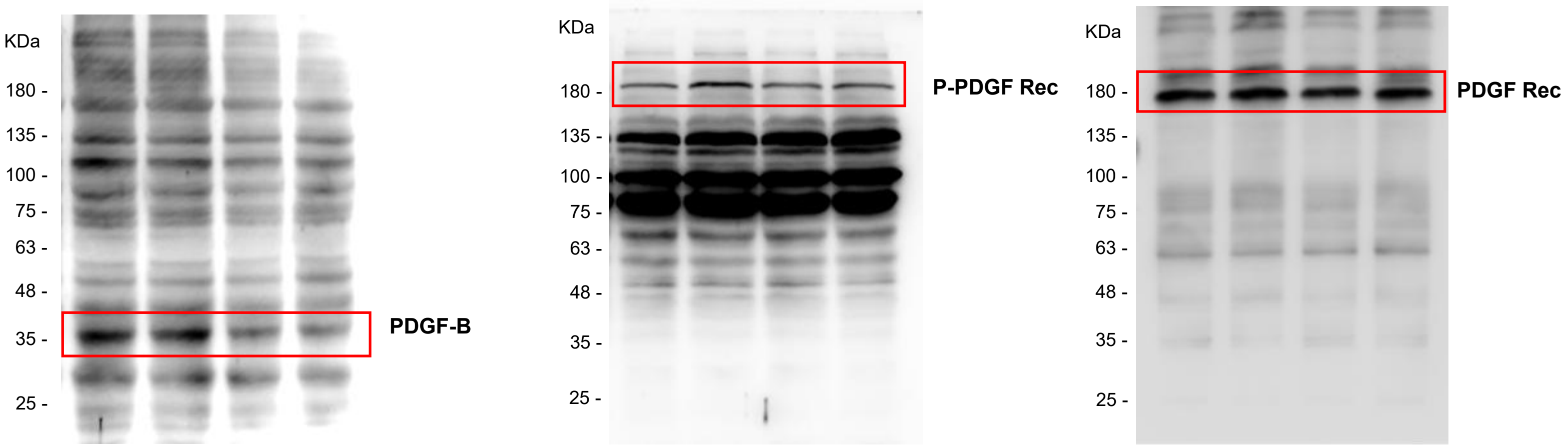

Fig S6 c

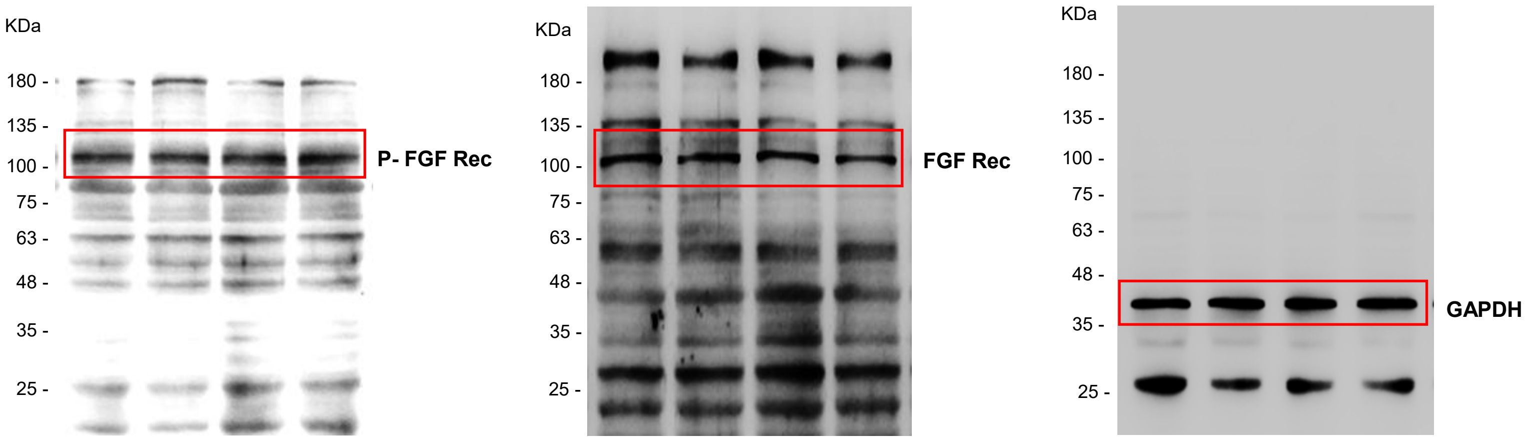

Fig S6 d

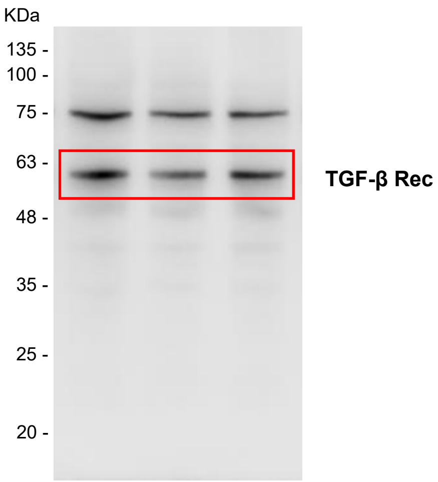

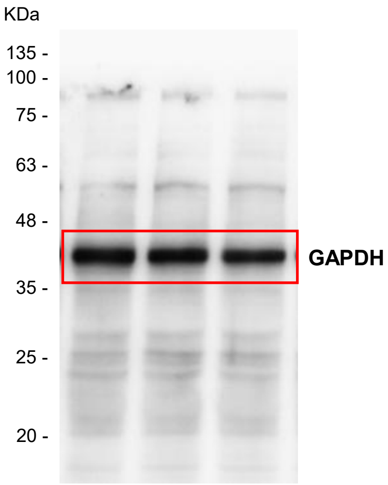

Fig S8

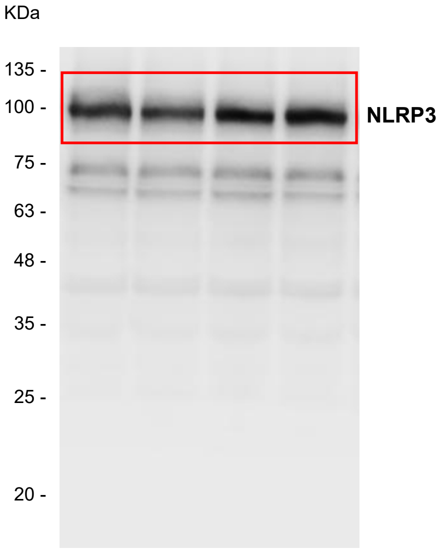

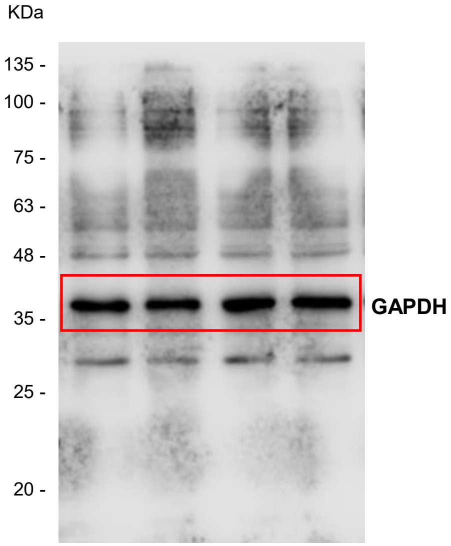

Supplement: Supplementary file 1 [file antioxidants-15-00912-s001.zip › antioxidants-4355498-supplementary.pdf]
